# Orbital-Selective Instabilities and Spin Fluctuations at the Verge of Superconductivity in Interlayer-Expanded Iron Selenide

**DOI:** 10.1021/acs.chemmater.5c01488

**Published:** 2025-10-21

**Authors:** Alexandros Lappas, Myrsini Kaitatzi, Alexandros Deltsidis, Izar Capel Berdiell, Laura Simonelli, Alexander Missyul, Martin Etter, Emil S. Bozin

**Affiliations:** † Institute of Electronic Structure and Laser, 124215 Foundation for Research and Technology−Hellas, Vassilika Vouton, Heraklion 71110, Greece; ‡ Department of Materials Science and Engineering, University of Crete, Voutes, Heraklion 70013, Greece; § 430128 ALBA Synchrotron Light Source, Carrer de la Llum 2-26, Cerdanyola Del Vallés 08290, Spain; ∥ 28332 Deutsches Elektronen-Synchrotron (DESY), Notkestraße 85, Hamburg 22 607, Germany; ⊥ Center for Solid State Physics and New Materials, Institute of Physics Belgrade, University of Belgrade, Pregerevica 118, Belgrade 11080, Serbia; # Condensed Matter Physics and Materials Science Division, Brookhaven National Laboratory, Upton NY 11973, United States

## Abstract

Understanding electron correlation-driven instabilities and their coupling to structural phases is essential for deciphering multiorbital pairing in unconventional superconductors. We investigate Li_*x*_(C_5_H_5_N)_*y*_Fe_2_Se_2_ (*x* ∼ 0.6; *y* ∼ 0.7–0.9), a tetragonal β-FeSe intercalate with a superconducting transition temperature (*T*_c_ = 39 K) closely tied to an expanded Fe-layer spacing (∼11.4 Å). High-resolution synchrotron X-ray diffraction and core-level absorption spectroscopy reveal subtle lattice distortions on cooling without a symmetry-breaking transition. Instead, the material exhibits negative thermal expansion (NTE) in the two-dimensional Fe network below *T*_S_ ∼ 70 K, and stiffening of local Se–Fe–Se bond dynamics near *T*_c_. The spatially incoherent rearrangement of FeSe_4_ tetrahedra and the site-local fluctuations, signal reduced electron correlations compared to those of parent β-FeSe (*T*_c_ = 8 K). Complementary X-ray emission spectroscopy, a fast local probe of Fe 3*d* valence states, detects persistent local Fe spin moments below *T*_S_, unlike quenching in related systems. These findings indicate that decoupling of Fe planes leads to an electronically driven lattice instability. The latter emerges as NTE induced from weak, orbital-selective localization of in-plane Fe 3*d* states rather than conventional transverse vibrations. Governed by Hund’s coupling, this selectivity permits coexistence of local spin fluctuations with itinerant *d*-electrons—critical for enhancing *T*_c_. These results suggest that intercalation-driven *d*-orbital differentiation moderates electron correlations, providing a pathway to optimize the superconductivity in low-dimensional quantum materials.

## Graphical abstract

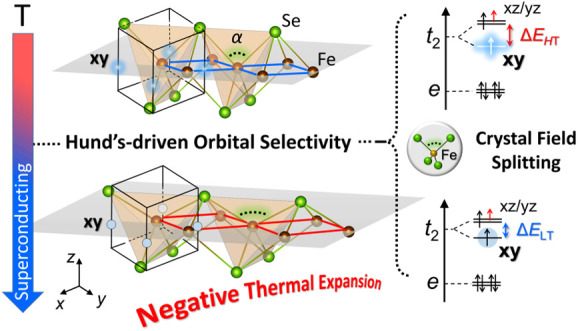

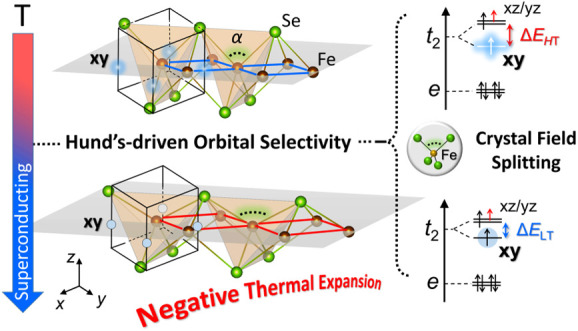

## 1 Introduction

Iron-based superconductors (FeSCs) are two-dimensional (2D) materials with high critical transition temperatures (*T*_c_ ≤ 65 K), driven by strong electronic correlations.^1^ Their Fe plane underpins a five-orbital model, where a multiband electronic structure creates electron and hole pockets at the Fermi surface, enabling unconventional pairing.^2^ The multiorbital nature of FeSCs is strongly influenced by Hund’s coupling, which enhances orbital differentiation and independence.^3^ This results in varying electron correlations across Fe 3*d* orbitals,^4,5^ with the *d*_*xy*_ exhibiting stronger localization than the *d*_*xz*_/*d*_*yz*_ ones.^6,7^ Strong electron–electron interactions amplify this effect, promoting an orbital-selective Mott phase (OSMP) in highly correlated systems.^8^ OSMP, marked by *d*-orbital degeneracy lifting, appears in various partially filled *d*-electron systems, where selected orbitals may order^9,10^ or fluctuate,^11^ profoundly affecting material properties. The overall behavior poses a key question: how strong must correlations be for high-*T*_c_ superconductivity?

The effects are nontrivial in Fe-chalcogenides (FeChs)^12^ that exhibit stronger electron correlations than Fe-pnictides,^6,7^ despite similar electron counts (∼6 per Fe). Since correlation strength depends on structural parameters like the Fe–Ch–Fe pathway,^6,13^ FeChs provide an ideal platform for studying orbital-dependent correlations. In these materials, strong correlations often induce symmetry-breaking electronic orders, raising the question of their competition with superconductivity, especially under doping.^14^ A key example is the nematic state in β-FeSe, where a tetragonal (*C*_4_) to orthorhombic (*C*_2_) transition (*T*_nem_ < 90 K)^15^ lifts *d*-orbital degeneracy without magnetism. While electronic orbital ordering (OO) is debated as its cause,^16^ the lattice responds to nematic fluctuations via nematoelastic coupling,^17^ linking structural changes to orbital occupancy shifts.

The intriguing electron correlation aspects of FeChs, linked to spin and orbital orders,^18^ necessitate new experimental platforms to clarify their role in superconductivity. The parent β-FeSe (*T*_c_ = 8 K) is ideal for such studies due to its tunable orbital-selective Cooper pairing^19^ via pressure,^20^ reduced dimensionality (e.g., monolayers),^21^ or intercalation of large organic molecules into the van der Waals (vdW) gap.^22^ Intercalation tunes structures from 3D to 2D, revealing a strong link between *T*_c_ and Fe-plane separation (*d*).^23^ When *d* exceeds ∼8.6 Å, the Fermi surface becomes 2D,^24^ and *T*_c_ no longer follows a simple linear dependence on *d*. In this regime, increased interlayer separation reduces electronic screening while enhancing Hund’s coupling-induced orbital-selective correlations, crucial for pairing instability.^25^ Understanding how electron doping via intercalation drives correlation-induced instabilities is key to exploring the *T*_c_ limit (∼46 K) at extreme *d* values.

The hybrid high-*T*_c_ superconductor Li_*x*_(C_5_H_5_N)_*y*_Fe_2–*z*_Se_2_ is developed as an experimental platform to study these phenomena.^26,27^ Co-intercalation of pyridine (C_5_H_5_N = Py) and Li, followed by annealing, doubles the Fe-sheet separation (*d* ≈ 11.2 Å) and raises *T*_c_ to ∼39 K—five times that of β-FeSe.^28^ However, identifying here the leading order parameter—in spin, orbital, or lattice channels that breaks the *C*_4_ symmetry—remains a challenge as lattice and interorbital fluctuations are intertwined. This arises from FeSe_4_ tetrahedral distortions that affect orbital occupancy (e.g., vertical elongation reduces *d*_*xy*_ electron hopping)^7^ and magnetic interactions,^13^ central to unconventional pairing. Understanding the crystal structure’s response to intercalation and superconductivity is crucial. Here, synchrotron X-ray probes provide insights into subtle distortions via (i) powder diffraction for Rietveld and pair distribution function (PDF) analyses of global and local structures and (ii) site-selective X-ray absorption (XAS)^29^ and emission (XES) spectroscopies to analyze Fe 3*d* electron interactions,^30^ orbital splittings, and local spin moments on time scales of electron dynamics (∼fs).^31^

The research strategy employs two complementary methods to elucidate local structure modifications and their connection to emerging electronic effects: (i) PDF analysis of the 300 K data, exploring the structure across multiple length scales, including the local regime; (ii) extended X-ray absorption fine structure (EXAFS) analysis, a subset of XAS, to track the evolution of the local structure across the studied temperature range. The two probes yield a consistent picture, namely, that local orthorhombicity, induced by electronic nematic fluctuations, as in the parent compound,^15^ can be ruled out and a tetragonal local structure is a valid description for the intercalated phase. Besides, room-temperature PDF analysis (see Section 3.1 below) shows that intercalation results in (i) compression of FeSe_4_ tetrahedra, adopting an anion height (*h*_*z*_ ∼ 1.45 Å) that deviates from those at the empirically determined *T*_c_ summit (cf., *h*_*z*_ ∼ 1.38 Å in pnictides,^32^ and *h*_*z*_ ∼ 1.50 Å in chalcogenides^33^), and (ii) induction of disordered FeSe_4_ distortions over longer length scales. Temperature-dependent studies (see Section 3.2 below) investigate this disorder. Rietveld refinements (see Section 3.2.1 below) reveal fine details at *T*_S_ = 70 K and *T*_c_ = 39 K, in the absence of a global symmetry-breaking transition. At *T*_S_, negative thermal expansion (NTE) appears in the Fe 2D network on cooling with increasing anisotropic microstrain broadening with respect to in-plane lattice directions. Approaching *T*_c_, abrupt changes in thermal expansion coefficients (TECs) suggest coupling of the microstrain to superconductivity. While NTE has been seen before in layered superconductors,^34^ here it does not correlate with *T*_c_. Instead, a sign change of in-plane TEC on cooling below *T*_S_ is observed, suggesting contraction at a rate of about 3–4 times larger than in ternary metal oxides, where conventional transverse vibrational motion typically drives such a behavior.^35^ Alongside the highly negative TECs (cf., −27.7 × 10^– 6^ K^–1^ at ∼39 K), among the highest in Fe-based systems,^36^ anharmonic atomic rearrangements that are linked to a potential electronically or magnetically^34^ induced structural instability are motivated by intercalation (see Section 3.3 below).

The drawn picture indicates that physics at unexplored short length scales drives global effects. Core-level spectroscopies at the Fe K-edge (see Section 3.2.2 below) are thus crucial for detecting subtle electron configuration changes that induce local lattice distortions.^30^ X-ray absorption near-edge structure (XANES), a subset of XAS, reveals that intercalation reduces Fe-ligand orbital hybridization with sharper changes at *T*_c_, indicating a redistribution of 3*d* orbitals. In support, EXAFS shows that Fe–Fe and Fe–Se local atomic displacements respond to superconductivity, evident in local mode hardening on cooling across on *T*_c_. Complementary Fe Kβ XES (see Section 3.3.1 below) adds on the electronic origin of the local lattice changes. It reveals a fluctuating Fe-3*d* local magnetic moment (μ) below *T*_S_—surprisingly differing from μ quenching in related FeChs.^37,38^ The electronic behavior is attributed (see Section 3.3.2 below) to a Hund’s coupling-driven incoherent-to-coherent crossover in the in-plane *d*_*xy*_ orbitals.^4,6^ This process leads to weak electronic anisotropy and contributes to an unusual NTE at low temperatures. In this context, the emerging coupling of itinerant *d*-electrons and fluctuating local spin moments at the Fe sites provides a rationale^39^ relevant for the elevated *T*_c_ in the intercalated phase.

The work emphasizes that variations in the involvement of specific Fe-3*d* orbitals—achieved by structurally tuning the system to selectively adjust hopping within individual Fe-orbital characters (e.g., *d*_*xy*_)—play a key role in moderating electron correlations away from the strongly correlated regime of β-FeSe. This effect may be a significant contributor to spin-fluctuation-mediated interactions at large interlayer separations, where an enhancement in *T*_c_ is observed.

## 2 Experimental Details

### 2.1 Synthesis and Characterization

High-quality single crystals of β-Fe_2–*z*_Se_2_ were synthesized from Fe 5N and Se 4N reagents in a stoichiometric 1.1:1.0 molar ratio by chemical vapor transport, using a eutectic mixture of anhydrous KCl 2N and AlCl_3_ 5N as the transport agent.^40^ The crystals were pulverized and utilized in subsequent intercalation reactions. Polycrystalline samples of the intercalated derivative Li_*x*_(C_5_H_5_N)_*y*_Fe_2–*z*_Se_2_ were synthesized with a modest-temperature (80 °C) solvothermal method under anaerobic conditions.^26,27^ In a typical reaction, β-Fe_2–*z*_Se_2_ powder and 3N Li pieces, in a 1:1 molar ratio, were stirred with anhydrous 2.8N pyridine in a 20 mL crimped vial, where the [Li:Py] solution molarity was adjusted to *M* = 0.2. The as-made sample was then loaded in a quartz ampule (⌀ 5 mm), sealed under vacuum (10^–2^ mbar), and heated at 180 °C for 2 days, after which it was air-quenched to produce the annealed derivative. All manipulations of these highly air-sensitive materials were undertaken inside an Ar-gas circulating MBRAUN (UNILab) glovebox, with <1 ppm of O_2_ and H_2_O. The phase purity of the as-made and annealed materials was verified by X-ray powder diffraction (Cu-Kα; Bruker D8 Advance). Temperature-dependent AC magnetic susceptibility measurements (*H*_ac_ = 1 Oe, *f* = 999 kHz; Figure S1) were obtained using an Oxford Instruments MagLab EXA 2000 vibrating sample magnetometer and a Quantum Design MPMS XL7 SQUID magnetometer. In this study, state-of-the-art synchrotron X-ray experiments were pursued with Li_*x*_(C_5_H_5_N)_*y*_Fe_2–*z*_Se_2_ samples synthesized using 0.3 mol of Li per mole of FeSe, with pyridine as the solvent. This corresponds to a nominal lithium content of *x* ∼ 0.6. The composition was selected based on previous studies,^26,27^ which investigated the optimization of material synthesis by varying the nominal Li content from *x* ∼ 0.2 to 1.0. These studies found that while the *T*_c_ was maximized across all Li contents, the presence of impurity phases was minimized near *x* ∼ 0.6.

### 2.2 High-Resolution Synchrotron X-ray Diffraction

Powder diffraction experiments for the intercalated, annealed Li_*x*_(C_5_H_5_N)_*y*_Fe_2–*z*_Se_2_ derivative were performed at the BL04-MSPD beamline of the ALBA light source (λ = 0.6193 Å) utilizing the Multi-Analyzer Detector (MAD) setup in high angular resolution mode.^41^ Powder samples were securely sealed inside airtight Kapton tubes (⌀1 mm) and loaded in a continuous-flow He-cryostat, allowing for sample spinning. Patterns were obtained over the range 20 ≤ *T* ≤ 300 K to provide a systematic structure assessment in both the normal and superconducting states. Exploration for a good candidate structural model was performed on the basis of the difference Fourier map analysis using the TOPAS software suite^42^ (Section S1.2). Quantitative information on the temperature evolution of the average structure was extracted after refinement of the established model by the Rietveld technique, utilizing the GSAS-II suite^43^ (Section S1.3). Relevant crystal structure drawings were produced by VESTA.^44^

### 2.3 High-Energy Synchrotron X-ray Diffraction

In order to investigate the impact of intercalation on the local atomic structure, complementary total scattering measurements on Li_*x*_(C_5_H_5_N)_*y*_Fe_2–*z*_Se_2_ at 300 K were obtained from the P02.1 beamline of the PETRA III synchrotron radiation source at DESY (λ = 0.20735 Å). A two-dimensional (2D) Varex XRD 4343CT flat panel detector was used at two different sample-to-detector distances (SDD), namely, 280 mm (near) and 2300 mm (far), determined by a LaB_6_ (NIST660c) calibrant. For comparison, the reference, parent β-Fe_2–*z*_Se_2_, was also measured at 300 K in the P21.1 beamline of PETRA III (λ = 0.12203 Å), with a 2D PerkinElmer XRD1621 area detector, at SDD: 357 mm and SDD: 1509 mm, determined by calibrating to a sample of known lattice parameter (Ni). The two detector locations offer quantifications from a broad *Q*-range, appropriate for pair distribution function (PDF) analysis, and from a higher-angular resolution, suitable for medium-resolution Rietveld analysis. For the 2D data reduction, the DIOPTAS suite^45^ was utilized to generate the 1D XRD patterns (i.e., Intensity vs *Q*). Afterward, PDFgetx3 ^46^ was employed to produce the sample-dependent structure factor *S*(*Q*), the reduced structure factor *F*(*Q*), and finally the total scattering *G*(*r*) functions, that is $G\left(r\right)=\frac{2}{\pi }\int_{0}^{\infty }Q\left[S\left(Q\right)-1\right]\;\sin\left(Qr\right)\;dQ$ (*Q*_max_ = 21.9 Å^–1^). Ultimately, the PDFgui suite^47^ provided quantitative local structure assessment by fitting the *G*(*r*)­s with appropriate structural models suggested by the Rietveld analysis (Section S2).

### 2.4 Synchrotron X-ray Core-Level Spectroscopy

The Fe (*E*_0_ = 7112 eV) K-edge XAS measurements were performed in transmission mode at the beamline BL22-CLÆSS of the ALBA synchrotron.^48^ The emitted X-rays were monochromatized using a Si(111) double-crystal monochromator with Rh-coated mirrors used to reject higher harmonics. Polycrystalline samples were mixed with BN (4N) in order to optimize the absorption jump at the Fe K-edge and pressed in ⌀5 mm pellets that were mounted in a continuous-flow He cryostat where the temperature was maintained within ±1 K through the 20–295 K range. Several scans were collected for any given temperature to ensure reproducibility and to improve the signal-to-noise ratio. XAS data normalization and modeling (Section S4.1) were performed with the ATHENA and ARTEMIS software suites, respectively.^49^ Qualitative assessment of the XANES region (Section S4.1.1) offered insights on the local electronic structure changes, while modeling of the EXAFS region, in the context of the single-scattering approximation (Section S4.1.2), offered quantitative information on the nearest-neighbor environments. The Fe Kβ XES measurements were performed in backscattering vertical geometry with the CLEAR emission spectrometer.^50^ The spectrometer utilizes a diced Si(333) dynamically bent analyzer crystal and a position-sensitive Mythen detector. The samples in powder form were filled into the ⌀2 mm holes of an airtight Al-holder and mounted in a He-flow cryostat. The Fe Kβ emission was measured by exciting the sample well above the Fe K-edge, at base (20 K) and high (295 K) temperatures with a high-resolution of ∼1.5 eV. Complementary, T-dependent (20–295 K) XES spectra were recorded with lower energy resolution (∼4.0 eV). These were normalized with respect to the integrated intensity in the energy range 7029–7079 eV. Quantitative information (Section S4.2) from the spectral variations across different samples and temperatures was extracted by means of the integrated absolute difference method (IAD).^51^

## 3 Results and Discussion

### 3.1 Complementary Length-Scale Views of Intercalation

#### 3.1.1 Average Structure

Indexing of the high-resolution synchrotron X-ray powder diffraction patterns (λ = 0.6193 Å) suggest that the high-*T*_c_ annealed derivative retains a tetragonal average structure all the way down to 20 K. Specifically, Le Bail full-profile fittings confirm that the *I*4/*mmm* symmetry (*a* = *b* = 3.8044(1) Å, *c* = 22.7945(4) Å) is favored after intercalation, rather than *P*4/*nmm* observed in the parent β-FeSe (Figure 1a and Section S1.1). Difference Fourier map analysis within the TOPAS suite^42^ offered a good candidate crystallographic model (Section S1.2 and Figure S1). The model was refined with the Rietveld method against the synchrotron data (Figure 1b,c) to allow comparisons against the parent phase (Section S1.3 and Table 1). Upon intercalation, the inorganic layers acquire the *c*-axis stacking sequence of the ThCr_2_Si_2_, 122 structure type^52^ instead of the PbFCl, 111 structure type^53^ (Figure 1d). The model with the pyridine molecules, in an orientationally disordered configuration in-between the close-to-stoichiometric Fe–Se sheets after refinement, suggests a composition of Li_*x*_(C_5_H_5_N)_*y*_Fe_2_Se_2_ (*x* ∼ 0.6; *y* ∼ 0.9 ± 0.1).

**Figure 1:**
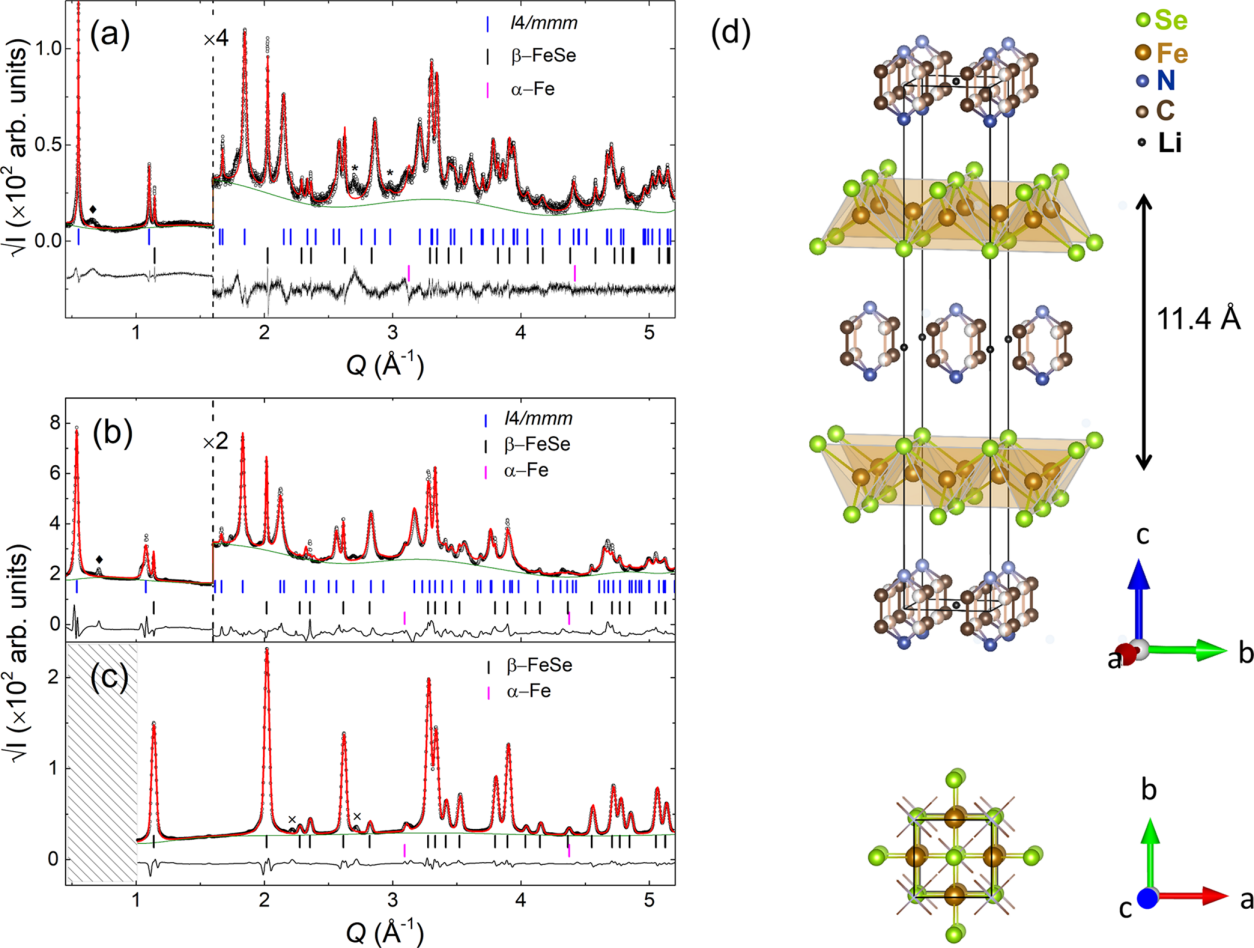
(a) Le Bail full-profile refinement of the high-resolution synchrotron X-ray powder diffraction pattern of the Li_*x*_(C_5_H_5_N)_*y*_Fe_2_Se_2_ at 20 K (λ = 0.6193 Å; *R*_w_ = 14.45%, GOF = 2.14). Rietveld-refined synchrotron X-ray powder diffraction patterns measured at 300 K with a 2D detector for (b) Li_*x*_(C_5_H_5_N)_*y*_Fe_2_Se_2_ (*x* ∼ 0.6; *y* ∼ 0.9 ± 0.1), with SDD at 2300 mm (λ = 0.20735 Å; *R*_w_ = 10.91%, GOF = 17.25), and (c) parent β-FeSe, with SDD at 1509 mm (λ = 0.12203 Å; *R*_w_ = 6.85%, GOF = 3.14). The black points and red lines represent the data and calculated profile, respectively. The green curve is the background. The black line at the bottom is the difference between observed and calculated patterns. Tick marks depict the position of Bragg peaks for: (blue; *I*4/*mmm*) the intercalated lattice Li_*x*_(C_5_H_5_N)_*y*_Fe_2_Se_2_, (black; *P*4/*nmm*) the parent β-FeSe, (magenta; *Im3̅m*) the cubic phase of the α-Fe. (◆): marks layer stacking (*Q* ≈ 0.67 Å^–1^) at lower intercalant content. (∗): high-order (00*l*) reflections understated by Le Bail analysis lacking a preferred orientation model. (×): hexagonal (NiAs-type) FeSe minority phase. (d) Illustration of the Rietveld-refined structural model for the Li_*x*_(C_5_H_5_N)_*y*_Fe_2_Se_2_ expanded lattice (*d* ≈ 11.4 Å) phase. A probable orientationally disordered configuration of the intercalated molecule (C_5_H_5_N) is graphically presented by two intersected rings related by a center of symmetry and a 4-fold rotation along the *c*-axis; dark-colored atoms (C = brown, N = blue, H = not shown for simplicity) on one molecular orientation are superimposed on light-colored Py atoms on the other. Likely Li sites (•) were not part of the Rietveld-refined model (see text). Top view of the unit cell (*a*,*b* projection), with intersected pyridine molecules shown in wireframe style at the cell vertices.

**Table 1:** Summary of Crystallographic Parameters Derived from Rietveld Refinements of Medium- and High-Resolution Synchrotron XRDs for the Parent β-FeSe (λ = 0.12203 Å) and Expanded-Lattice Li_*x*_(C_5_H_5_N)_*y*_Fe_2_Se_2_ (*x* ∼ 0.6; *y* ∼ 0.7 ± 0.1) (*λ* = 0.6193 Å) Phases, Respectively (Details in Tables S1 and S2)

	β-FeSe	Li_*x*_(C_5_H_5_N)_*y*_Fe_2_Se_2_	
	*P*4/*nmm* ^a^,^c^	*I*4/*mmm* ^b^,^c^	
*T*/K	300	300	20
*a*,*b*/Å	3.7688(1)	3.8269(1)	3.8088(1)
*c*/Å	5.5162(2)	23.2066(5)	22.8209(4)
*V*/Å^3^	78.285(6)	339.86(2)	331.05(2)
*z*-Se/Å	0.2665(2)	0.3126(1)	0.3134(1)
Occupancy: Se	0.994(4)	0.994(1)	
*U*_iso, Se_/Å^2^	0.0096(2)	0.0109(5)	0.0072(5)
*U*_iso, Fe_ /Å^2^	0.0083(3)	0.0113(8)	0.0095(8)
Fe–Se/Å	2.3881(4)	2.402(1)	2.392(1)
Fe–Fe/Å	2.6649(1)	2.706(1)	2.693(1)
FeSe_4_ V_Td_/Å^3^	6.8524(1)	7.099(1)	6.746(1)
Se−Fe^−Se(a) /°	103.99(3)	105.59(7)	105.51(8)
Anion height, *h*_*z*_/Å	1.467(1)	1.452(2)	1.448(2)

a Atomic positions: Fe is located at 2*a* (0,0,0) and Se at 2*c* (0,^1^/_2_,*z*).

b Atomic positions: Fe is located at the 4*d* (0,^1^/_2_,^1^/_4_), Se at the 4*e* (0,0,z), N at the 4*e* (^1^/_2_,^1^/_2_, *z*), and C at the 16*m* (0.72,0.72,*z*) sites.

c Atomic positions: Fe-site occupancy was refined and found to be stoichiometric within the esd.

To rationalize the chosen Py configuration, it is worth noting that pyridine, as an aromatic N-heterocycle with sufficient electron affinity, reacts with alkali metals to form monovalent radical anions [C_5_H_5_N·−] via electron transfer to its LUMO (π^∗^).^54^ Studies further show that lithium atoms can interact with the aromatic π-system, coordinating above the plane of the ring—either centrally or at the edges—to form adducts.^55^ In the case of Li_*x*_(C_5_H_5_N)_*y*_Fe_2_Se_2_, such interactions likely govern the organization of Li–Py adducts within the van der Waals gaps, in contrast to the [Li–NH_3_]-intercalated superconducting analogs where π-coordination is absent.^56^ Although Li is not directly observable in this study, its likely placement at crystallographic sites 2*b* (0,0,^1^/_2_) or the less optimal 4*c* (0,^1^/_2_,0) may be supported by structural analogy (Section S1.4, Figure S1d,e). Consequently, the N-heterocyclic molecules are expected to coordinate on either side of Li, favoring a *C*_2_-axis orientation perpendicular to the Fe–Se sheets (Figure 1d). In this configuration, pyridine accepts additional electron density from Li, increasing the basicity of its nitrogen site, and its ability to participate in electron transfer via a Lewis acid–base interaction. This arrangement, with the lone pair on N, directed toward the Fe–Se layers, enables (i) effective coupling with the host’s electronic states, facilitating charge transfer, and (ii) a change from the primitive (*P*) to body-centered (*I*) Fe–Se layer stacking upon intercalation.

#### 3.1.2 Local Structure

Complementary assessment of short-range structural modifications was performed by using synchrotron X-ray total scattering. A qualitative evaluation of the 300 K structure–function, *F*(*Q*), of Li_*x*_(C_5_H_5_N)_*y*_Fe_2_Se_2_ (λ = 0.20735 Å) compared to the β-FeSe (λ = 0.12203 Å), reveals a sinusoidal diffuse signal at high-*Q* in both data sets (inset, Figure 2a,b), indicating disorder that is more pronounced in the intercalated phase (Section S2.1). This periodic signal in the *F*(*Q*) translates into a pronounced enhancement of the nearest-neighbor (NN) peak intensity in the *G*(*r*) (i.e., Fe–Se bond; *r* = 2.411(4) Å) relative to peaks at higher-*r* (Figure 2c,d), reflecting strongly bonded local building blocks that are less strongly coupled at longer distances.

**Figure 2:**
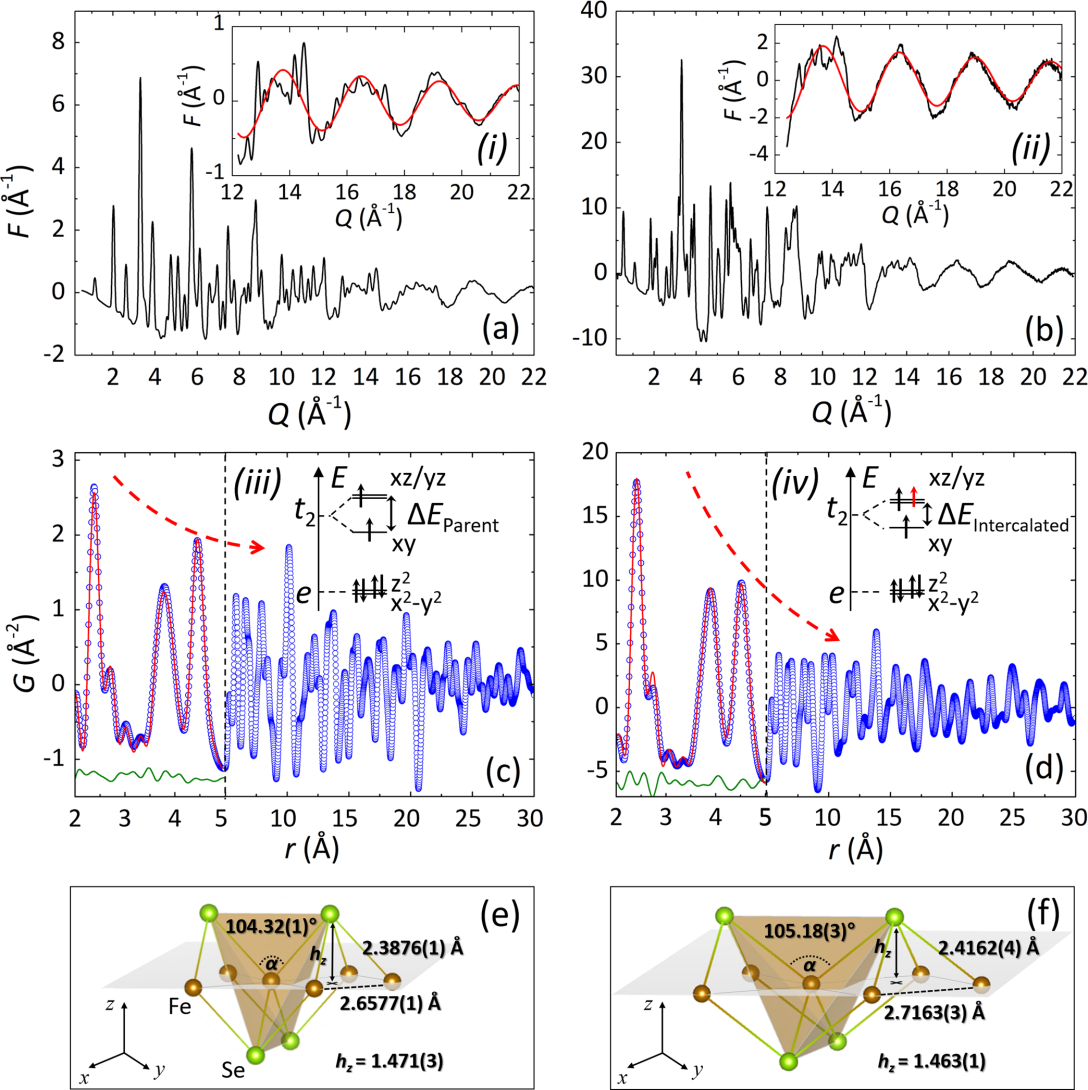
Reduced total-scattering structure functions, *F*(*Q*), for (a) the parent β-FeSe (SDD at 357 mm; λ = 0.1220 Å) and (b) the Li_*x*_(C_5_H_5_N)_*y*_Fe_2_Se_2_ (SDD at 280 mm; λ = 0.2074 Å) at 300 K. Insets: (i, ii) Fitting (red curves; see text) of the sinusoidal diffuse signal at high-*Q* (12 ≤ *Q* ≤ 22 Å^–1^). Experimental *G*(*r*) functions with the low-*r* PDF fits (2 ≤ *r* ≤ 5 Å; see text) of (c) the parent β-FeSe (*Cmma*) and (d) the Li_*x*_(C_5_H_5_N)_*y*_Fe_2_Se_2_ (*I*4/*mmm*); observed data (circles), small-box model (red traces), and their difference (green traces, offset for clarity). Dashed red lines are guides to the eye, highlighting the damping of the *G*(*r*). (e,f) Graphic of distorted FeSe_4_ tetrahedral geometry in the two systems, comparing local-scale modifications derived from PDF analysis; bond angle (α) and anion height (*h*_*z*_) from the Fe-plane. Insets: (iii, iv) Schematic of the crystal field splitting energy level diagram of the Fe 3*d* ^6^ ion in a tetrahedral coordination. Nonbonding valence shell electrons are depicted by black arrows, and the intercalation-driven electron doping is depicted by a red arrow. The deviation of the FeSe_4_ motif from ideal geometry relates to a tetragonal field that separates the *xy* from *xz/yz* type of 3*d* orbitals by Δ*E* ≈ 109.5 – α (Δ*E*_Parent_ > Δ*E*_Intercalated_).

For quantitative analysis via PDF, the tetragonal model (cf., *I*4/*mmm* space group) suggested by Rietveld analysis (Table S1) was used as the starting point (Section S2.2). It provides a good description of the intermediate *G*(*r*) *r*-range (*r* = 2–40 Å) for β-FeSe (*R*_w_ ∼ 7%; Figure S2a), but performs poorly for the intercalated (*R*_w_ ∼ 19%; Figure S3a). The inferior PDF fit for the intercalated compound reflects significant structural disorder that the model cannot adequately capture, whereas Rietveld refinements account for this through the inclusion of microstrain along different crystallographic directions (see Section 3.2.1 below and Section S3.2). This discrepancy arises because Rietveld analysis probes the average long-range order, while PDF analysis is sensitive across multiple length scales, including local structure.

As short-range distortions typically manifest in the low-*r* range, further PDF analysis focused on the nanoscale range (*r* ≤ 10.5 Å). This approach was motivated by earlier studies on β-FeSe, where the tetragonal model at room temperature exhibits a subtle misfit in the *G*(*r*) at *r* ∼ 3.6–4.0 Å.^57^ This feature has been attributed to inequivalent NN Se–Se pair distances, previously interpreted as evidence of electronic local nematicity in which *C*_4_ symmetry is locally broken. Following this concept, a symmetry-breaking *C*_4_-to-*C*_2_ local orthorhombic distortion (cf., *Cmma* space group), assuming possible short-range correlations, was also considered for the current PDF analysis (Sections S2.2 and S2.3). The orthorhombic model for Li_*x*_(C_5_H_5_N)_*y*_Fe_2_Se_2_ (Figures S4a,b; *r* = 2–9 Å) performs poorly because it attempts to broaden the feature at *r* ∼ 3.6 Å by introducing additional inequivalent Se–Se distances, as would be expected for locally broken symmetry (cf., β-FeSe). The region *r* = 2–5 Å is particularly informative: local orthorhombicity provides a good description of *G*(*r*) for β-FeSe (*R*_w_ ∼ 5.5%; Figures 2c and S2b,c), but yields a statistically poorer fit (*R*_w_ ∼ 8.0%) of the intercalated phase. In contrast, the tetragonal model accurately describes the intercalated phase (*R*_w_ ∼ 5.2%; Figure S4c,d). Since the experimental data show no evidence of peak broadening at *r* ∼ 3.6 Å, the tetragonal local structure model—with the fewest adjustable parameters—is favored for the intercalated phase (Figure 2d).

The sensitivity of the latter model on the broadening and rapid damping of the *G*(*r*) peak intensities at far-neighbor atomic pairs has been further assessed. Quantitative “box-car” PDF fits (Section S2.2 and Figure S3b–f) point to the presence of likely incoherent local domains^58^ in the Li_*x*_(C_5_H_5_N)_*y*_Fe_2_Se_2_ structure, with a spatial extent of about ∼1.2 nm, as indicated by the rise of the *R*_w_ (Figure S3b). The relevant structural parameters derived from PDF analyses are compiled in Figure 2e,f. Elongation of pair distances, with squashed along the *c*-axis FeSe_4_ units, was found upon intercalation. These motifs regulate the Fe 3*d* ^6^ orbital occupancy and magnetic interactions,^13^ with the separation (Δ*E*) of *d*_*xy*_ and *d*_*xz*_/*d*_*yz*_ orbitals being sensitive to the Se–(Fe)–Se bond angle (α). As this scales with the FeSe_4_ unit’s deviation from the ideal 109.5° geometry (cf., Δ*E* ∼ 109.5 – α),^59^ the differentiation between *d*_*xy*_ and the other *t*_2_ orbitals (*d*_*yz*_, *d*_*xz*_) is expected to be somewhat reduced upon intercalation (Δ*E*_Parent_ > Δ*E*_Intercalated_; inset, Figure 2c,d). Overall, the PDF analysis indicates that a tetragonal-to-orthorhombic distortion, with short-range correlations due to electronic, local nematicity, as observed in β-FeSe at room temperature,^57^ is not evident under the present experimental conditions in the intercalated system. The observed disorder in the local structure is therefore likely to have a different origin (see Section 3.3 below).

### 3.2 Structural Modifications across the Critical Temperature

#### 3.2.1 Global Lattice Effects

While the room-temperature analysis implies structural imperfections, changes in the average structure with temperature speak for the role of electronic effects (e.g., orbital occupation and magnetic interactions) leveraged by intercalation. This provides a strong incentive to obtain experimental evidence in view of the likely coupling of the lattice to electronic degrees of freedom as the correlated superconducting state is approached.

Sequential Rietveld refinements of the Li_*x*_(C_5_H_5_N)_*y*_Fe_2–*z*_Se_2_ structure reveal key details at two characteristic temperatures: *T*_S_ = 70 K and *T*_c_ = 39 K. Below *T*_S_, a puzzling negative thermal expansion (NTE) occurs in the electronically active Fe-square plane $\left(a=b=\sqrt{2}\times \mathrm{Fe-}\mathrm{Fe}\right)$ , despite an overall cell-volume contraction (inset, Figure 3a). This effect is not attributable to an obscure global symmetry-lowering *C*_4_-to-*C*_2_ transition (Section S3.1 and Figures S5, S6) characteristic of nematicity,^15^ conferring the inadequacy of the specific electronic process as a driver. To accurately describe the NTE, (*hkl*)-dependent peak-shape variations in the powder patterns at high-*Q* (Figure S7) are incorporated into the analysis. Their quantification using the Stephens phenomenological model of anisotropic peak broadening^60^ allows to characterize the distribution of microstrain along different crystallographic directions^61^ (Section S3.2 and Figure S8). The refined crystallographic parameters suggest close-to-stoichiometric Fe–Se sheets and a composition of Li_*x*_(C_5_H_5_N)_*y*_Fe_2_Se_2_ (*x* ∼ 0.6; *y* ∼ 0.7 ± 0.1) (Table S2).

**Figure 3:**
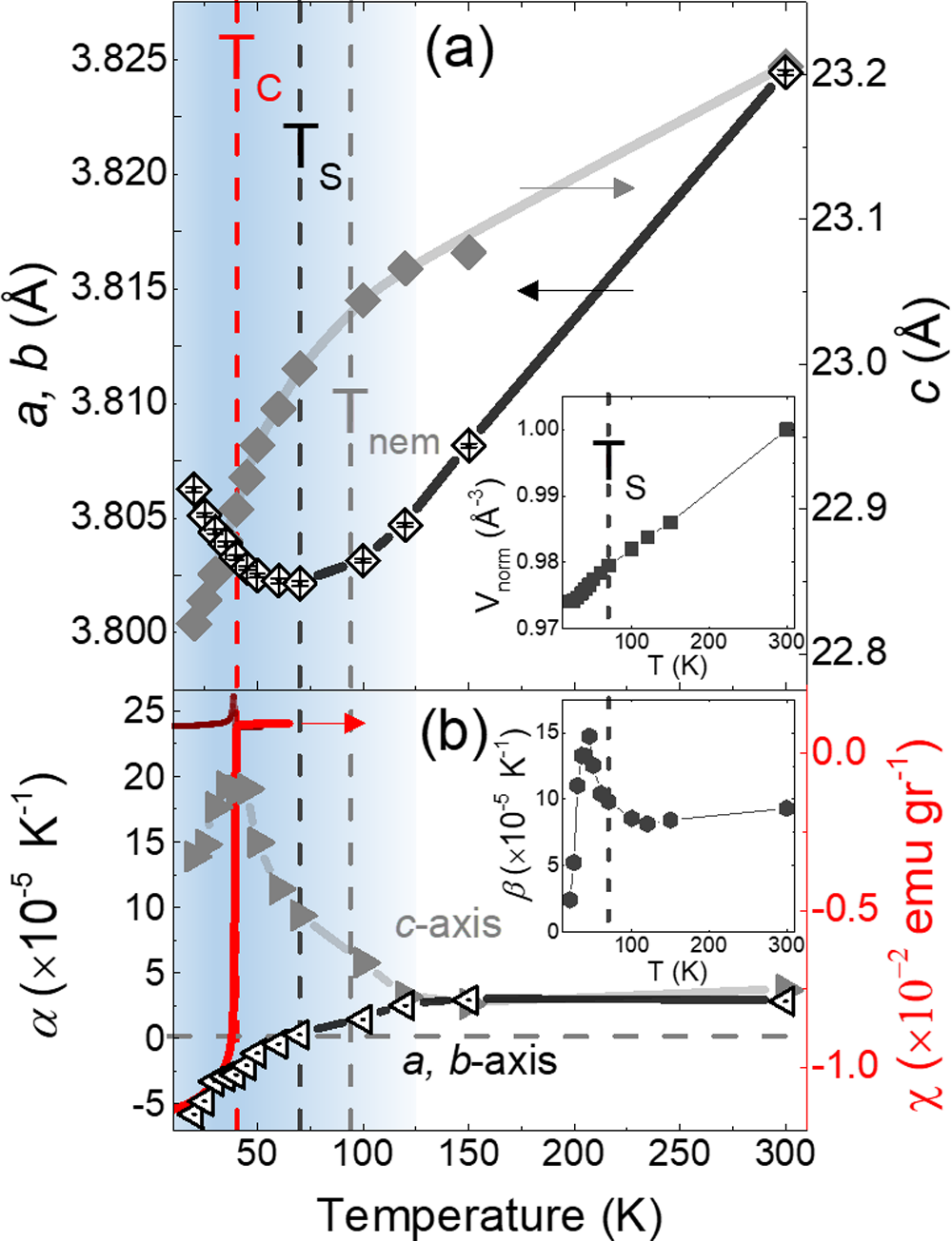
Temperature evolution of the Li_*x*_(C_5_H_5_N)_*y*_Fe_2_Se_2_ (*x* ∼ 0.6; *y* ∼ 0.7 ± 0.1), (a) Rietveld-refined lattice parameters (*a*,*b*-plane: open diamonds; *c-*axis: filled diamonds). Inset: unit cell volume (filled squares), normalized to 300 K, and (b) (left) linear thermal expansion coefficients (TECs, see text; *a*,*b*-axis: open triangles, *c*-axis: filled triangles) and (right) AC susceptibility (*H*_ac_ = 1 Oe, *f* = 999 Hz; χ′, red line; χ″, dark red line). Inset: volume TEC (filled hexagons). Vertical dashed lines mark the onsets of: the superconducting critical temperature, *T*_c_; the Fe-square plane negative thermal expansion (NTE) onset, *T*_S_; the global *C*_4_-to-*C*_2_ transition met in the β-FeSe nematic state, *T*_nem_. The shaded region: depicts the temperature scale for the development of global lattice effects.

Notably, when in-plane lattice directions, relevant to the Fe 2D network, where NTE occurs, contribute to the anisotropic microstrain peak broadening, the magnitude of the *S*_*hkl*_ terms progressively elevates; namely, from minimal for out-of-plane to maximal when both basal plane directions are incorporated (Figure 4). Collectively, the evolution of the *S*_*hkl*_ parameters and the enhanced in-plane anisotropy of microstrain (Figure S8) establish a microscopic connection with local atomic rearrangements that lack coherence for a global distortion. However, the anomalies at *T*_S_ suggest that electron–lattice interactions become significant on cooling toward the superconducting state. Given the multiorbital nature of the system, the redistribution of crystal field splitting levels (inset, Figure 2c,d)—particularly the predominantly in-plane *d*_*xy*_ orbital relative to the other *t*_2_ orbitals (*d*_*yz*_, *d*_*xz*_)—may be relevant to the observed structural changes.

**Figure 4:**
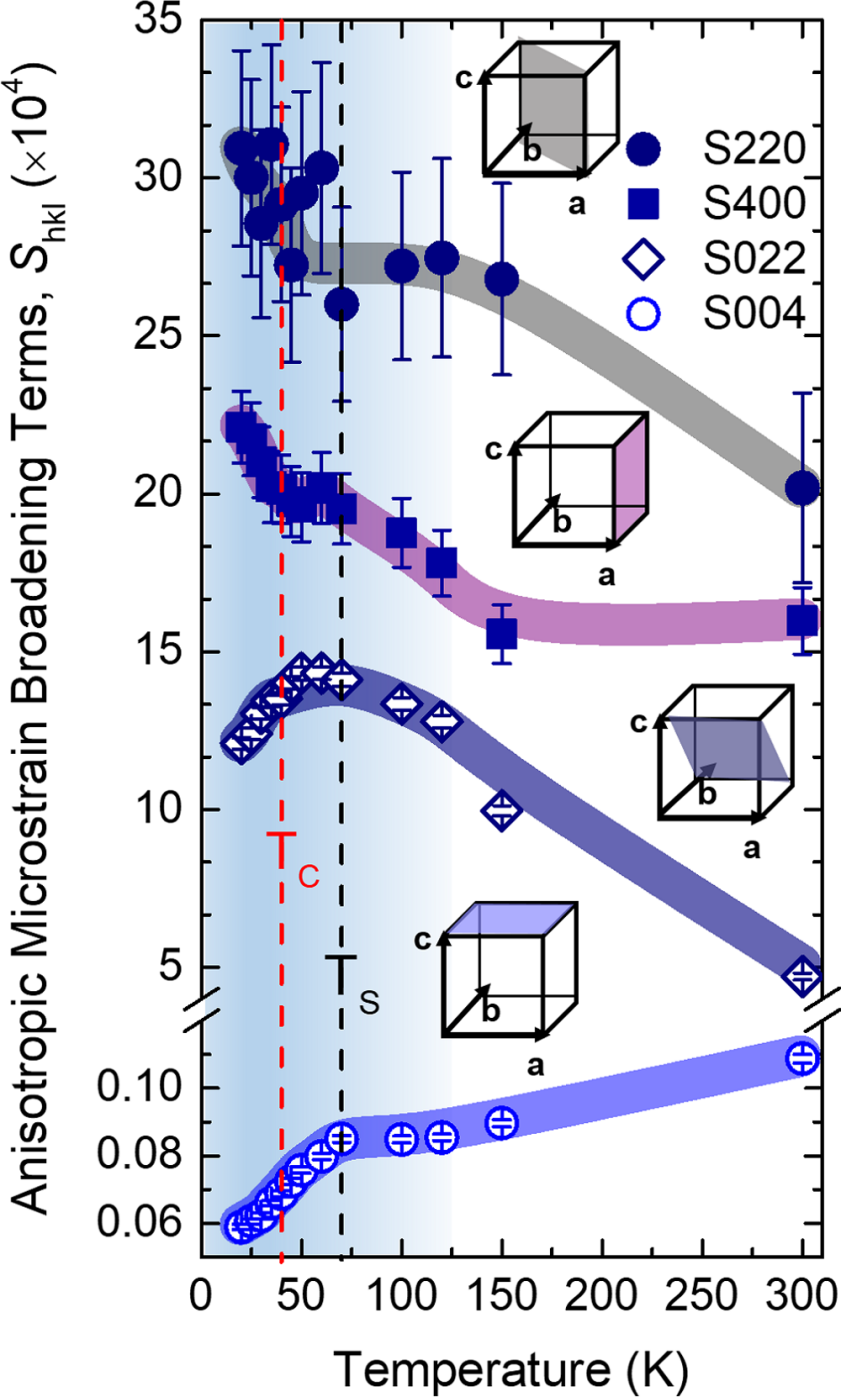
Temperature evolution of the (*hkl*)-dependent anisotropic peak broadening Stephens coefficients, *S*_*hkl*_. Vertical dashed lines mark the onsets of the superconducting state (*T*_c_) and the Fe-square plane negative thermal expansion (*T*_S_). The broad lines over the data are guides for the eye, highlighting regions of steeper evolution for the *S*_*hkl*_ parameters. Cubes depict graphically, in color-shading, planes normal (±) to specific lattice directions (cf., [001], [011], [100], [110]) where microstrain develops.

Based on these observations, the lattice thermal expansion coefficients (TECs; Section S3.3) were calculated. The linear TECs, α_α_ (Figure 3b), become negative for *T* < *T*_S_, while α_c_ exhibits a peak at *T*_c_. Meanwhile, the volume TEC, β (inset, Figure 3b), shows a sharp maximum at *T*_c_ before collapsing. Such anisotropic thermal expansion behavior, characteristic of layered structures with strong in-plane chemical bonding,^62^ is also observed in other layered superconductors of varying chemical compositions, such as La_1.85_Sr_0.15_CuO_4_,^63^ MgB_2_,^64^ and Ba­(Fe_1–*x*_Co_*x*_)_2_As_2_,^65^ where the NTE onset may be linked to *T*_c_. The lattice response at *T*_c_, as reflected in the TECs, has been interpreted in terms of Gibbs free energy changes in the superconducting phase,^34^ which associate spontaneous lattice strain with the order parameter. In Li_*x*_(C_5_H_5_N)_*y*_Fe_2_Se, this is evidenced by the alignment of the β­(*T*) variation (Figure 3b, inset) with the steeper evolution of *S*_*hk*l_ for *T* < *T*_c_ (Figure 4). However, the precise nature of the subtle sensitivity of *S*_*hkl*_(*T*) around *T*_S_ where NTE sets in remains unclear.

#### 3.2.2 Local Bonding Correlations

The context of the global structure findings associates microstrain with the superconducting order parameter and motivates questions on whether physics at unresolved, shorter length scales plays a role in the electronic properties of such a multiorbital system. Thus, beyond the global effects, the responses of the local structure to (i) the intercalation of the molecular spacer layer and (ii) the superconductivity are sought. Relevant geometrical parameters of the FeSe_4_ units (Section S3.4) that have been debated to enable *T*_c_ parametrization,^32,66^ suggest that the tetrahedra become squashed, in accordance with the local Fe–Se layer becoming thinner (cf., *h*_*z*_ compressed) on moving from the parent to the intercalated phase (Table 1 and Figure S9). In that respect, element-selective, Fe K-edge XAS becomes instructive in probing sensitive local Fe–Se sheet distortions with local sensitivity on the femtosecond (fs) time scale.^29^

Inspection of the XANES region shows a small diminution of the spectral weight in the pre-edge peak #A (∼7112 eV) upon intercalation (Figure S11 and Section S4.1.1), implying a modest decrease in the Fe 3*d* and Se 4*p* orbital mixing,^67,68^ in agreement with the FeSe_4_ geometry modification. Upon cooling through *T*_c_, a noticeably steeper change in the intensity of peak #A indicates a redistribution of 3*d* states, likely due to electron density depletion from the bonding Fe–Se pair to the Fermi level as a result of Cooper pairing. Since instantaneous atomic distortions (departures from tetragonality) driven by electronic nematic fluctuations may occur outside the dynamic response window accessible to PDF, EXAFS—as a subset of XAS, being a site-selective probe with local sensitivity on the fs time scale—was employed to detect possible signatures underrepresented in PDF (Section 3.1.2). Quantitative information from the analysis of Fe K-edge EXAFS (Figure S12 and Section S4.1.2) reveals the evolution of the local structure across the studied temperature range. Figure 5a shows two-shell model fits of the Fourier transforms of representative EXAFS oscillations that are described well by the tetragonal Li_*x*_(C_5_H_5_N)_*y*_Fe_2_Se_2_ lattice. Under the present experimental conditions, EXAFS modeling of the intercalated system consistently supports the absence of local orthorhombicity induced by nematic fluctuations similar to β-FeSe (Figure S13 and Section S4.1.3). EXAFS further confirms, in agreement with PDF at 300 K, that the local geometrical parameters relative to β-FeSe follow similar trends: (i) elongation of NN Fe–Se and Fe–Fe distances, (ii) compression of anion height, *h*_*z*_, and (iii) widening of bond angle, α (Table S3).

**Figure 5:**
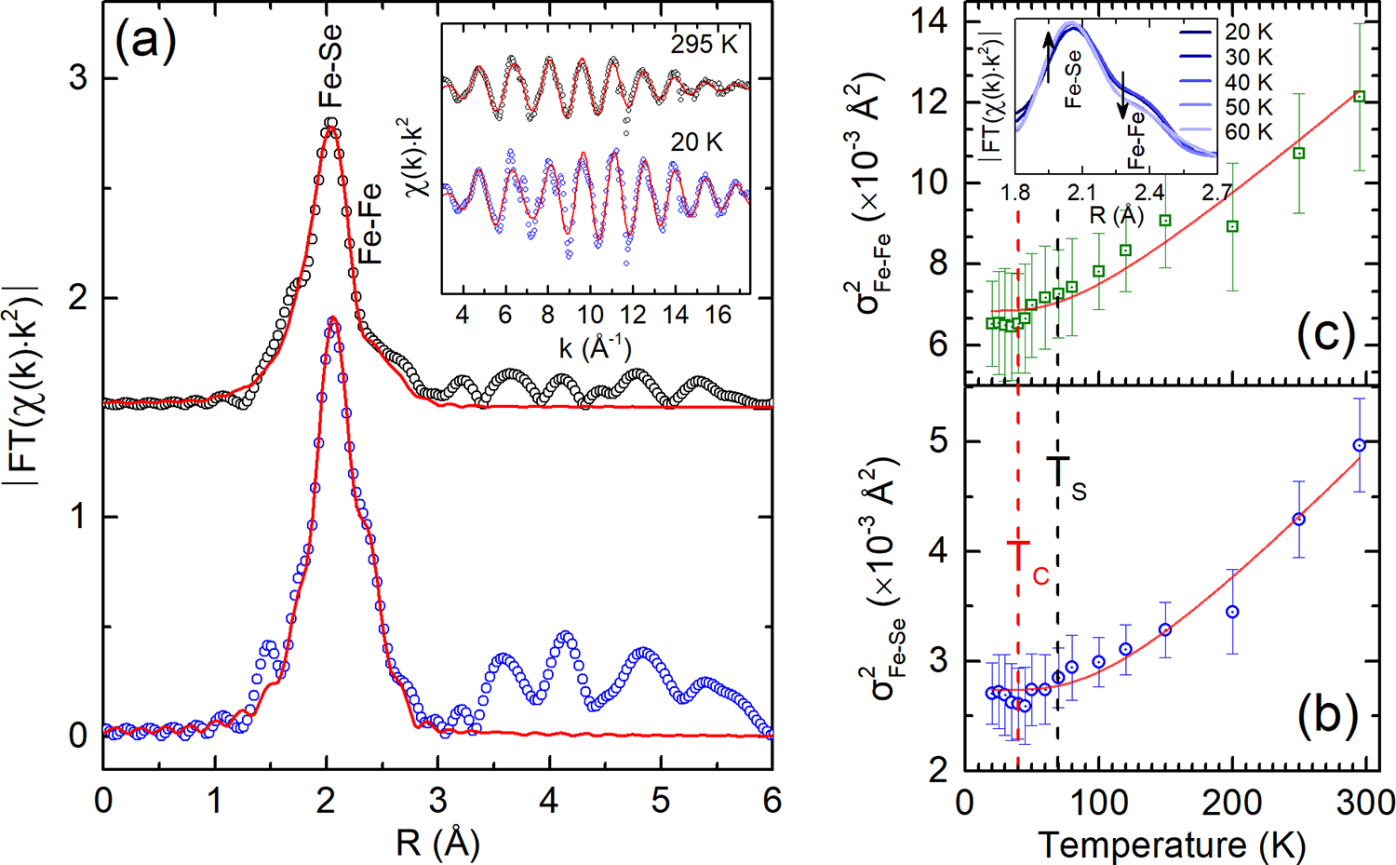
For the Li_*x*_(C_5_H_5_N)_*y*_Fe_2_Se_2_ compound, (a) representative two-shell model fits (solid line; see text) of the Fourier transforms (FT) of EXAFS oscillations at 20 K (blue circles) and 295 K (black circles). Inset-a: The corresponding EXAFS signals with the *k*-space fitting. Temperature evolution of the MSRDs (σ^2^) for the local Fe–Se (b) and Fe–Fe (c) atomic distances. The solid red lines depict the fitting with the correlated Einstein model (50 ≤ *T* ≤ 295 K), extrapolated below *T*_c_ to mark the deviation from the data. Inset-c: FT of EXAFS data across *T*_c_ identifying near-neighbor Fe–Fe and Fe–Se pair-distance modifications; arrows depict a subtle “discontinuity” in the evolution of the intensities across *T*_c_ that manifest as changes in bond dynamics (σ^2^).

Small changes though, can be better picked up via the local bond dynamics measured by the mean-square relative thermal displacements (σ^2^; correlated Debye–Waller factors) of a pair of atoms. Figure 5b,c compiles the T-dependence of σ^2^ for the Fe–Se and Fe–Fe near-neighbor distances. This provides direct information on the dynamic lattice distortions and offers the Einstein temperature, *θ*_E_, describing the respective bond stiffness (cf., $k=\mu \omega_{E}^{2}$ ; Section S4.1.4). From the σ^2^(*T*) between 50 and 295 K, the *θ*_E_ was determined, as 341 ± 8 K and 259 ± 8 K, for the Fe–Se and Fe–Fe distances, respectively. This differing behavior of the local-scale metrics is comparable to that in the binary β-FeSe (∼318 ± 5 K and ∼263 ± 5 K)^27^ and the prototype molecule-intercalated Li_*x*_(NH_3_)_*y*_Fe_2_Se_2_ (313 ± 10 K and 248 ± 10 K).^69^

Interestingly, for the Py-intercalated derivative, σ^2^(*T*) deviates from the correlated Einstein-like behavior. This is marked by a delicate but systematic downturn at *T*_c_ (Figure 5b,c) that appears as a subtle “discontinuity” in the evolution of the peak intensity (Figure 5c, inset) corresponding to the Fe–Fe (Fe–Se) distances when the sample is cooled from above the *T*_c_ to well below. The effect is weaker for the Fe–Se than Fe–Fe distances, as the former appears harder and with a lower static disorder than the Fe–Fe (Table S4). In view of the anomalies in the local bond dynamics, σ^2^(*T*) and the *S*_hkl_(*T*) near *T*_c_, it is reasonable to propose that microstrain (cf., *S*_400_ and *S*_220_) in the electronically active Se–Fe–Se sheets controls the local lattice fluctuations, especially since strain (or pressure) is known to sensitively adjust the superconducting *T*_c_.^34^ The decrease in the instantaneous local lattice distortions at *T*_c_, witnessed by σ^2^(*T*), has also been observed in the atomic correlations measured in diverse by nature, intermetallic,^70^ cuprate,^71^ and molecule-intercalated Fe-selenide^69^ superconductors. The presence of this anomaly, indicating a local-mode hardening upon cooling across *T*_c_, provides extra evidence for the connection between electron–lattice interactions and superconductivity in Li_*x*_(C_5_H_5_N)_*y*_Fe_2_Se_2_.

### 3.3 Electronically Induced NTE

The evolution of dynamic local lattice distortions in the electronically active Se–Fe–Se layers of Li_*x*_(C_5_H_5_N)_*y*_Fe_2_Se_2_ appears to be connected with changes in thermal expansion at *T*_c_ (Figure 3b). However, the NTE in the Fe–Fe planar network does not directly link to *T*_c_, raising the question whether electronic effects, beyond a purely structural mechanism (e.g., transverse vibrations), play a role.^72^ NTE in Fe-based phases often emerges near an instability when tuning a parameter like carrier doping,^73^ which shapes complex phase diagrams where structural, spin, orbital, and superconducting order parameters interact.^14^ A striking example is Ca_1–*x*_La_*x*_Fe_2_As_2_,^36^ where doping induces anharmonic lattice vibrations,^74^ likely due to electronic and magnetic fluctuations coupled to the lattice. This brings a structural instability that results in exceptionally large in-plane NTE (e.g., α_α_^max^∼ – 41 × 10^–6^ K^–1^, *x* = 0.15; *T* = 75 K).^36^ Similarly, in Li_*x*_(C_5_H_5_N)_*y*_Fe_2–*z*_Se_2_, the in-plane TEC changes sign below *T*_S_ (70 K) (Figure 3b), reaching large negative values (α_α_ = −27.7 × 10^–6^ K^–1^; *T* = 39 K), among the highest in Fe-based systems. Inspired by La-doped CaFe_2_As_2_, it is plausible that intercalation-induced charge doping brings this hybrid superconductor also close to an electron correlation-driven instability.

#### 3.3.1 Electronic Structure Changes at Short Timescales

In such Fe-based phases, the Mott–Hund’s framework^1^ predicts that itinerant and local Fe 3*d* electrons act as independent degrees of freedom, emerging on short time scales. Consequently, site-local fluctuations in the charge or spin channels—potentially driving lattice distortions—can occur more rapidly than the response time of conventional structural probes, and may therefore be masked due to time-averaging effects.^75^ XES then, as a fast probe with local sensitivity at the femtosecond (fs),^30,31^ plays a pivotal role in exploring this scenario. It enables the detection of local fluctuations and provides insights into the multiorbital electronic structure, potentially revealing whether an incipient electron correlation instability underlies the observed NTE.

Fe Kβ XES spectra were acquired at both low (Figure 6) and high (Figure S14) spectral resolutions. The interaction between Fe 3*d* ^6^ valence electrons and the 3*p* ^5^ core–hole in the final state gives rise to a strong Kβ_1,3_ peak and a broader Kβ′ shoulder at lower energies.^76^ The energy separation between Kβ′ and Kβ_1,3_ is approximately Δ*E* = *J*(2*S* + 1) (inset, Figure 6a), where *J* is the exchange integral between the 3*p* and 3*d* wave functions, and *S* represents the total spin of the unpaired 3*d* electrons.^51^ Since the 3*p*–3*d* interaction is inherently local, XES serves as a direct probe of the local spin moment (μ) within the Fe 3*d* shell,^31,77^ reflecting short time scales (Section S4.2). The weak intensity of the Kβ′ feature is indicative of a low-spin (LS) configuration (Fe^2+^ $e^{4}t_{2}^{2}$ , *S* = 1).^68^ A key feature observed at both spectral resolutions is a subtle shift of the Kβ_1,3_ peak to lower energy upon cooling below *T*_c_ to 20 K (see difference curve, Figure S14). In Li_*x*_(C_5_H_5_N)_*y*_Fe_2_Se_2_, this behavior suggests a higher fluctuating Fe μ at room temperature compared to that at base temperature. This trend is characteristic of FeSCs, where localized magnetism in the normal state is suppressed in the superconducting state by strong quantum fluctuations driven by electron itinerancy.^75^

**Figure 6:**
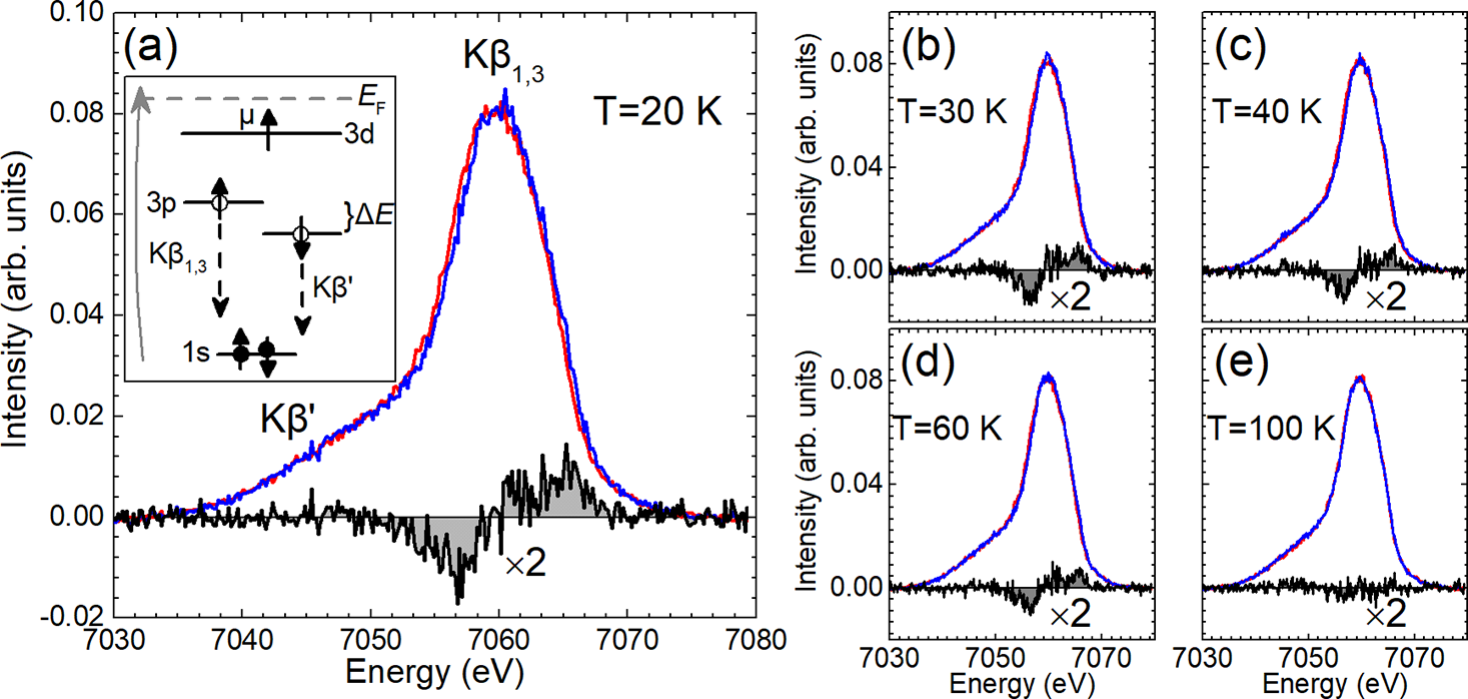
(a) Comparison of the Fe Kβ emission line of Li_*x*_(C_5_H_5_N)_*y*_Fe_2_Se_2_ at 20 K (blue) with respect to the reference β-FeSe measured at 100 K (red). Their spectral difference (black) is also shown underneath. Inset: Schematic diagram of the Kβ emission process. Two final states, Kβ_1,3_ and Kβ’, with opposite core–hole spin, are generated due to the intra-atomic interaction of the 3*p* core hole with the net magnetic moment μ in the 3*d* valence shell; filled and open circles depict electrons and holes, respectively. (b–e) Evolution of XES for Li_*x*_(C_5_H_5_N)_*y*_Fe_2_Se_2_ at representative temperatures, as portrayed in the difference plots derived by subtracting the 100 K XES spectrum of the reference β-FeSe, from those of the molecule-intercalated compound. The spectral differences are multiplied by two.

Low-resolution experiments offer additional insights into the temperature-dependent evolution of the local magnetic moment. By subtracting the 100 K XES spectrum of nonmagnetic β-FeSe from that of the intercalated compound (Figure 6), a relatively flat difference at 100 K suggests that the Fe local moment μ in the expanded lattice is comparable to that of the reference compound.^37,78^ However, a distinct evolution emerges upon cooling, manifested in Kβ spectral changes (Figures 6 and S15) quantified using the integrated absolute difference (IAD) method relative to a lower spin reference (Section S4.2).^51^ The IAD analysis reveals a fluctuating Fe μ in the 3*d* valence shell, which begins to increase near *T*_S_, coinciding with the onset of NTE, and persists through the superconducting state (Figure 7a). This behavior contrasts with the suppression of Fe μ below *T*_c_ observed in other intercalated FeChs, such as K_*x*_Fe_2–*y*_Se_2_ ^37^ and (Li_1–*x*_Fe_*x*_)­OHFeSe,^38^ which exhibit shorter interlayer distances (*d* < 9.3 Å).

**Figure 7:**
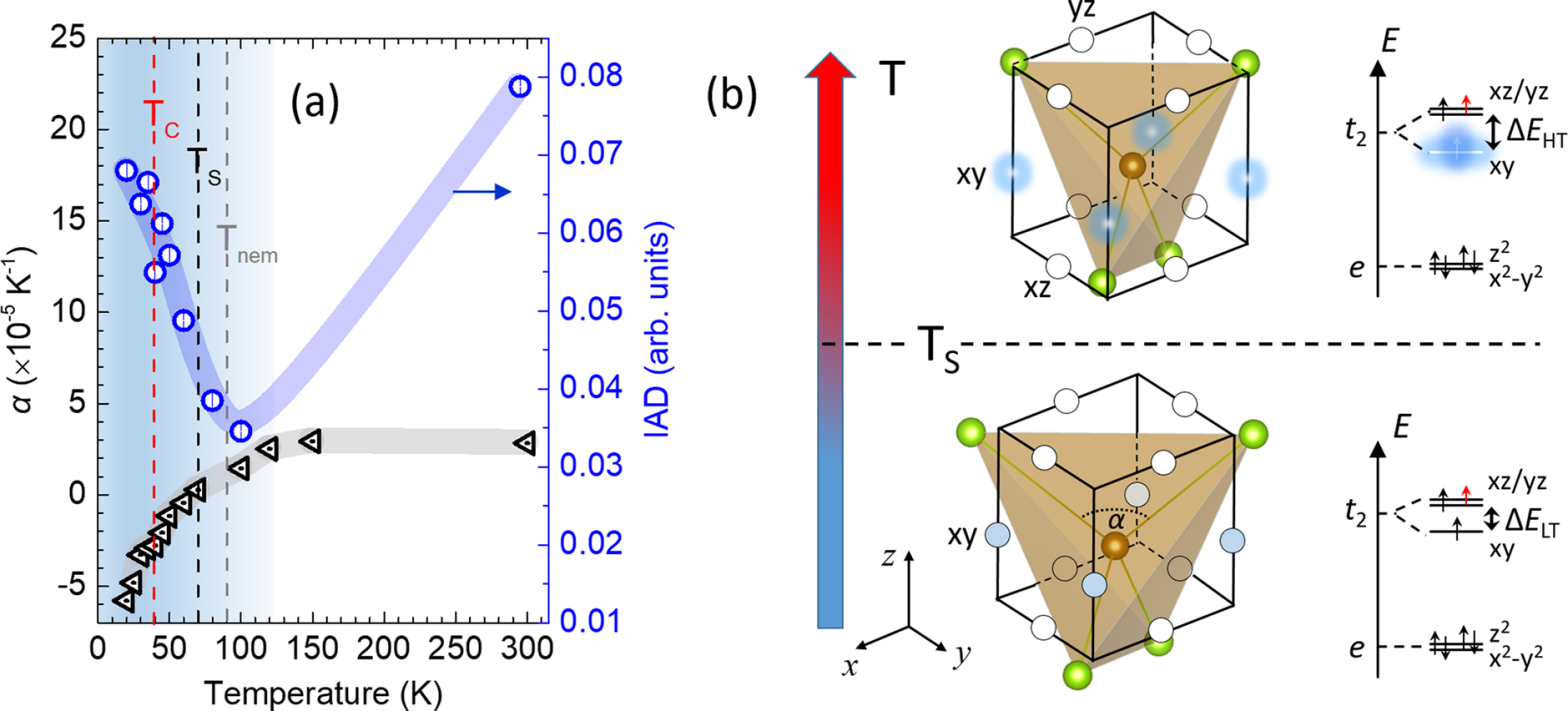
(a) Li_*x*_(C_5_H_5_N)_*y*_Fe_2_Se_2_, linear thermal expansion coefficient, α, for the Fe-square plane (triangles) from Figure 3b, and temperature evolution of the Fe Kβ XES IAD (circles); broad lines are guides to the eye. Dashed lines mark onsets of: superconducting critical temperature, *T*_c_; the Fe-square plane negative thermal expansion (NTE), *T*_S_; the global *C*_4_-to-*C*_2_ transition met in the β-FeSe nematic state, *T*_nem_. Shaded region: depicts the temperature scale for emerging fluctuating local spin moments. (b) Graphic arrangement of Fe 3*d* ^6^ ion *t*_2_ (*d*_*xy*_, *d*_*yz*_, *d*_*xz*_) orbitals and the relative energy diagram of nonbonding *d* electrons in a tetrahedral crystal field. *t*_2_ orbitals are directed toward the center of cube edges, with spheres resembling the center of gravity of regions with high electron density. The FeSe_4_ distortion away from ideal tetrahedral geometry (see Section S3.4) causes the separation of the *d*_*xy*_ from *d*_*xz*_/*d*_*yz*_ states (cf., Δ*E* ≈ 109.5 – α; Δ*E*_HT_ > Δ*E*_LT_); nonbonding valence shell electrons are populated as black arrows and electron doping is shown by a red arrow. Slightly elongated vs compressed FeSe_4_ units (along *c*-axis) denote Hund’s coupling-induced incoherent-to-coherent crossover (*T*_S_) on the 3*d*_*xy*_ orbital; marked by an itinerant (hazy clouds) to localized (filled spheres) character of electron charges (see text).

The pronounced increase in IAD, along with the enhancement of NTE in the Fe-plane below *T*_S_ (Figure 7a), suggests that the origin of the anomalous thermal expansion is closely linked to complex magnetic interactions. This behavior may be associated with a broader physical concept known as the “Invar effect”,^79^ which generally describes the very low (<2 × 10^–6^ K^–1^ at ∼300 K) or negative thermal expansion below the Curie (or Néel) temperature of certain alloys. In this context, a negative contribution to thermal expansion—arising from variations in the amplitude of local magnetic moments and spin fluctuations^34^—can compensate the positive contribution from lattice thermal vibrations. A key theoretical framework for understanding such anomalous thermal expansion is the spin fluctuation theory.^80^ This theory considers the dual character (cf., localization and itinerancy) of *d*-electron systems and incorporates electron–electron correlations within the itinerant electron model. While the latter may be valid for Li_*x*_(C_5_H_5_N)_*y*_Fe_2_Se_2_, with static magnetic ordering being absent, such a magnetism-induced NTE is disregarded, but the role of Fe-3*d* orbital-dependent interactions is worth examining as a cause of an electronically driven instability that couples to the lattice.

#### 3.3.2 Orbital-Selective Correlations

The XES findings and multiorbital nature of Li_*x*_(C_5_H_5_N)_*y*_Fe_2_Se_2_ motivate discussing the observations in the context of Hund’s coupling-induced orbital-selective correlations,^4^ which drive instabilities^10^ such as electronic nematicity.^15^ The possible activation of orbital-dependent correlations^13^ in the intercalated system is supported by a comparison of key structural parameters with those of the parent phase (Table 1 and Figure 2c,d). They reveal: (a) an elongated Fe–Se bond, consistent with increased electron doping,^28,81^ which moves the system away from half-filling, and (b) a less acute Se–(Fe)–Se bond angle (α) resulting in a compressed tetrahedron (shorter *h*_*z*_), which—beyond affecting the out-of-plane *d*_*xz*_/*d*_*yz*_ orbitals—also facilitates electron hopping between the in-plane *d*_*xy*_ orbitals. Together, these features^6,7^ create conditions for weaker electron correlations in the intercalated phase compared to β-FeSe. Consequently, orbital-selective correlations^1^ in Li_*x*_(C_5_H_5_N)_*y*_Fe_2_Se_2_ evolve through a thermal crossover, marked by weak orbital-dependent renormalization that sets in at *T* < *T*_S_ without a global symmetry-breaking transition (Section S3.1 ). This contrasts with β-FeSe, where stronger correlations are associated with a *C*_4_-to-*C*_2_ transition at *T*_nem_ < 90 K.^15^

While XRD provides a time-averaged perspective, XES offers complementary insights into the thermal crossover at *T* < *T*_S_ (Figure 7a). In the Mott–Hund’s framework, orbital renormalization involves a coherence-incoherence crossover.^4^ As temperature decreases, the coherence and spectral weight of the more localized *d*_*xy*_ orbitals increase, along with their hybridization with the itinerant *d*_*xz*_/*d*_*yz*_ orbitals.^8^ Due to the local nature of the core–hole potential, Fe Kβ XES primarily probes localized *d*_*xy*_ electrons, while itinerant electrons may be underrepresented.^78^ Here, the increase in IAD below *T*_S_ (Figure 7a) likely marks the temperature at which the growing local magnetic moment is associated with the increased coherence of electronic states, most likely due to selective localization on the in-plane Fe-*d*_*xy*_ orbitals. This trend is reminiscent of earlier NMR observations in β-FeSe, where an incoherent-to-coherent 3*d*_*xy*_ crossover at ∼*T*_nem_, driven by Hund’s coupling, was linked to on-site ferromagnetic exchange interaction between local and itinerant spins.^82^ A similar, albeit weaker, coupling appears in Li_*x*_(C_5_H_5_N)_*y*_Fe_2_Se_2_, leading to a fluctuating Fe μ below ∼*T*_S_.

Linking the orbital occupation to subtle structural changes clarifies the behavior in the crossover region. Electrons near *E*_F_ typically occupy nonbonding (or weakly antibonding) *d* states,^81^ making the interaction with bonding electron pairs relevant. Intercalation-driven interlayer expansion: (i) alters the Se–(Fe)–Se angle (α), reducing the *d*_*xy*_ – *d*_*yz*_/*d*_*xz*_ energy gap (Δ*E*) (inset, Figure 2c,d),^59^ and (ii) enhances Hund’s coupling,^25^ favoring unpaired *t*_2_ nonbonding electrons in the same atomic shell. Below *T*_S_, selective localization on *d*_*xy*_ increases the electron count in nonbonding orbitals (Figure 7b). This raises energy costs^83^ due to repulsion between nonbonding valence electrons of the central Fe atom and Fe–Se bonding pairs. Therefore, changes in the nonbonding electron configuration at *T* < *T*_S_ create an instability, weakening Fe–Fe distances, causing NTE. Above *T*_S_, delocalization of the *d*_*xy*_ orbitals reduces repulsions, and the Fe–Fe interatomic potential becomes more strongly bonding, ultimately enabling positive thermal expansion (Figure 3a) and relief of the microstrain in the Fe 2D network (Figure 4).

Thus, intercalation-induced doping in Li_*x*_(C_5_H_5_N)_*y*_Fe_2_Se_2_ moderates electron–electron interactions, driving the system toward instability due to a fluctuating orbital-selective state. At the temperature scale of *T*_S_, this state results in weak in-plane electronic anisotropy, which ultimately leads to an unusual Fe-based 2D network NTE at low temperatures, rather than a global *C*_4_-to-*C*_2_ transition observed in the nematic state of the more correlated β-FeSe. The results do not decisively rule out predictions^13^ that the interplay between itinerant electrons and fluctuating local moments—at an intermediate correlation strength, with weak orbital differentiation—could be the primary factor driving high-*T*_c_ in the intercalated phase.

## 4 Conclusions

The study explores electron correlation-driven instabilities near superconductivity in iron-based superconductors, focusing on the layered Li_*x*_(C_5_H_5_N)_*y*_Fe_2‑z_Se_2_ (*x* ∼ 0.6; *y* ∼ 0.7–0.9), a β-FeSe intercalate.

Intercalation results in thinner Se–Fe–Se layers, leading to a compressed FeSe_4_ tetrahedral geometry that moderates orbital differentiation. Structural features (e.g., bond angles) suggest weaker electron correlations in the intercalated phase compared to β-FeSe. Furthermore, with the expansion of the Fe-plane separation (*d* ∼ 11.2 Å), structural distortions emerge as the correlated state is approached. These distortions manifest, (i) below *T*_S_ (∼70 K) as negative thermal expansion (NTE) in the two-dimensional Fe-network, accompanied by enhanced microstrain broadening involving in-plane lattice directions, and (ii) on further cooling, as hardening of local bond dynamics across *T*_c_ (∼39 K). Global (XRD) and local (XAS) probes reveal incoherent FeSe_4_ rearrangements and site-local fluctuations, linking lattice distortions to the underlying electronic effects.

These structural insights raise questions about the impact of shorter length-scale physics on the electronic properties of the multiorbital system. The puzzle is addressed through X-ray emission spectroscopy (XES), a fast probe with local sensitivity on the femtosecond (fs) time scale. Emergent fluctuating Fe local spin moments are observed below ∼*T*_S_, unlike their quenching in related superconducting FeChs. Capturing such rapid electronic variations provides crucial evidence linking NTE to a correlation-driven instability. The NTE in the 2D Fe network is attributed to weak electronic anisotropy in the Fe-3*d* in-plane orbitals, which emerges at ∼*T*_S_ due to orbital-selective correlations—a key feature of Mott–Hund’s framework.

The hybrid superconductor is on the brink of an electron-correlation-driven instability, as evidenced by the interplay between fluctuating local spin moments and *d*-electrons in the superconducting state. In this emerging picture of intermediate correlation strength, these spin fluctuations may critically contribute to enhance the *T*_c_ of the intercalated phase. Intercalation sensitively tunes key parameters to optimize the superconductivity in systems with large interlayer separations, facilitating the design of materials with elevated *T*_c_.

## Supplementary Material



## Notes

Additional details on the 300 K average crystal structure analysis, total scattering qualitative assessments and quantitative PDF modeling; Rietveld analysis (symmetry-lowering, microstrain, thermal expansion coefficients, geometrical parameters) from variable-temperature synchrotron XRD; normalization and modeling of the XAS (XANES and EXAFS regions) data, including the analysis of the XES as a function of temperature (PDF)
