# Supplementary material for: Orbital-Selective Instabilities and Spin Fluctuations at the Verge of Superconductivity in Interlayer-Expanded Iron Selenide
Source: Chem Mater. 2025 Oct 21;37(21):8581–94. doi: 10.1021/acs.chemmater.5c01488 (PMC12613316; doi:10.1021/acs.chemmater.5c01488)
Supplement: Supplementary file 1 [file cm5c01488_si_001.pdf]

Supporting Information (SI)

Orbital-Selective Instabilities and Spin Fluctuations at the Verge of Superconductivity in Interlayer-Expanded Iron Selenide

Alexandros Lappas,[a,*] Myrsini Kaitatzi,[a,b] Alexandros Deltsidis,[a,b] Izar Capel Berdiell,[a] Laura Simonelli,[c] Alexander Missyul,[c] Martin Etter,[d] and Emil S. Bozin[e,f]

[a]*Institute of Electronic Structure and Laser, Foundation for Research and Technology–Hellas, Vassilika Vouton, 71110 Heraklion, Greece*

[b]*Department of Materials Science and Engineering, University of Crete, Voutes, 70013 Heraklion, Greece*

[c]*ALBA Synchrotron Light Source, Carrer de la Llum 2-26, 08290 Cerdanyola del Vallés, Spain*

[d]*Deutsches Elektronen-Synchrotron (DESY), Notkestraße 85, 22 607 Hamburg, Germany*

[e]*Center for Solid State Physics and New Materials, Institute of Physics Belgrade, University of Belgrade, Pregerevica 118, 11080 Belgrade, Serbia*

[f]*Condensed Matter Physics and Materials Science Division, Brookhaven National Laboratory, Upton, NY11973, USA*

* Email: lappas@iesl.forth.gr

Contents

S1. Average structure analysis at 300 K 3

S1.1 Indexing 3

S1.2 Structure solution 3

Figure S1. Crystal Structure 4

S1.3 Rietveld analysis 5

Table S1. 6

S1.4 On the possible organization of Li-Py in the interlayer space 7

S2. Total scattering 8

S2.1 Qualitative assessment 8

S2.2 Quantitative model-based assessment. 8

Figure S2. *P*4/*nmm vs. Cmma* PDF analysis of β-FeSe 10

Figure S3. 'Box-car' PDF assessments of $Li_x(C_5H_5N)_yFe_2Se_2$ 11

S2.3 Local orthorhombicity in $Li_x(C_5H_5N)_yFe_2Se_2$. 12

Figure S4. *I*4/*mmm vs. Cmma* PDF analysis of $Li_x(C_5H_5N)_yFe_2Se_2$ 13

S.3 Temperature-dependent Rietveld analysis 14

S3.1 Quest for symmetry-lowering global distortion 14

Figure S5. T-dependent Rietveld, high-*Q* features 15

Figure S6. Test for global symmetry-lowering in the intercalated lattice 16

S3.2 Anistropic microstrain peak broadening 17

Figure S7. Isotropic *vs.* anisotropic microstrain broadening 18

Figure S8. Anisotropic microstrain 19

Table S2. 20

S3.3 Thermal-expansion coefficients 21

S3.4 Geometrical parameters 22

Figure S9. T-evolution of geometrical parameters 23

S4. Core-level spectroscopy 24

S4.1 X-Ray Absorption Spectroscopy: normalization and modelling 24

Figure S10. Basic sample characterization 27

Figure S11. Normalized XANES spectra 28

Figure S12. EXAFS oscillations and Fourier Transforms 29

Figure S13. EXAFS search of deviations from tetragonality 30

Table S3. 31

Table S4. 31

S4.2 X-Ray Emission Spectroscopy: data analysis 32

Figure S14. Normalized XES spectra: high- *vs*. low- spectral resolutions 33

Figure S15. Normalized XES spectra: temperature evolution 34

S5. References 35

S1. Average structure analysis at 300 K

S1.1 Indexing

The synchrotron XRDs (**Figure 1**) suggest that the basic structure of the high-T_c $Li_x(C_5H_5N)_yFe_{2-z}Se_2$ derivative brings an interlayer distance of d=11.42 Å (Q= 0.55 Å^{-1}, λ= 0.6193 Å), as determined by the low angle *(00l)* Bragg reflections. With the interlayer distance in the low-T_c, parent β-FeSe being d= 5.6 Å (Q= 1.12 Å^{-1}, λ= 0.12203 Å), a relation of the FeSe sheet-decoupling and the five-fold increase of the T_c by intercalation may be inferred. The unique attributes of high- photon flux and resolution, of synchrotron X-rays permit indexing of the intercalated material's patterns with three phases, namely, a majority tetragonal one having a 4×-supercell of β-FeSe along the *c*-axis (i.e., expanded-lattice), with β-FeSe and *α*-Fe as minority phases. Subsequently, multiphase Le Bail analysis carried with GSAS-II,[1] suggested that the parent β-FeSe upon intercalation and post-annealing crystalizes in the tetragonal *I*4/*mmm* space group, all the way down to 20 K.

S1.2 Structure solution

To solve the crystallographic structure of the expanded-lattice $Li_x(C_5H_5N)_yFe_{2-z}Se_2$ derivative, we utilized the TOPAS suite.[2] We applied difference Fourier map analysis starting with the tetragonal *I*4/*mmm* space group that was obtained from the Le Bail fit (**Figure 1a**). It consists of refining the XRD intensities assuming no guests in the FeSe lattice, adopting the *I*4/*mmm* symmetry, and excluding the low-angle region (affected by the guests). After the first iteration, the scale factor was fixed. Then the entire range was refined including the calculation of the structure factor difference between the experimental data and the 'empty' cell (cf., without the molecule) model. From the difference Fourier map analysis, residual electron density clouds (**Figure S1a**) could be obtained that were assigned to the molecule halfway between the Se-Fe-Se slabs. Within the resolution of the current experiments, the difference Fourier analysis locates the N atom on the general site 4*e*, with the symmetry operator 4*mm*, and the C atom on the general site 16*m*, with the symmetry operator ..*m*. These symmetry elements propose an orientationally disordered pyridine (C_5H_5N= Py) configuration. Graphically this is depicted by two superimposed Py molecules related by a combination of symmetry operations, that is a center of symmetry and a 4-fold rotation along the *z*-axis (**Figures 1d and S1b**). In the subsequent crystallographic model utilized for the Rietveld analysis, the apparent disorder of the intercalated molecule was approximated by assuming a pyrazine ($C_4H_4N_2$) molecular structure that captures the nitrogen configurational disorder along the layer stacking sequence, as suggested by the Fourier map analysis. The final tetragonal *I*4/*mmm* structure treats the molecules and the FeSe layers as one motif and accounts for the superimposed orientationally-disordered molecular configurations.

Figure S1. Crystal Structure

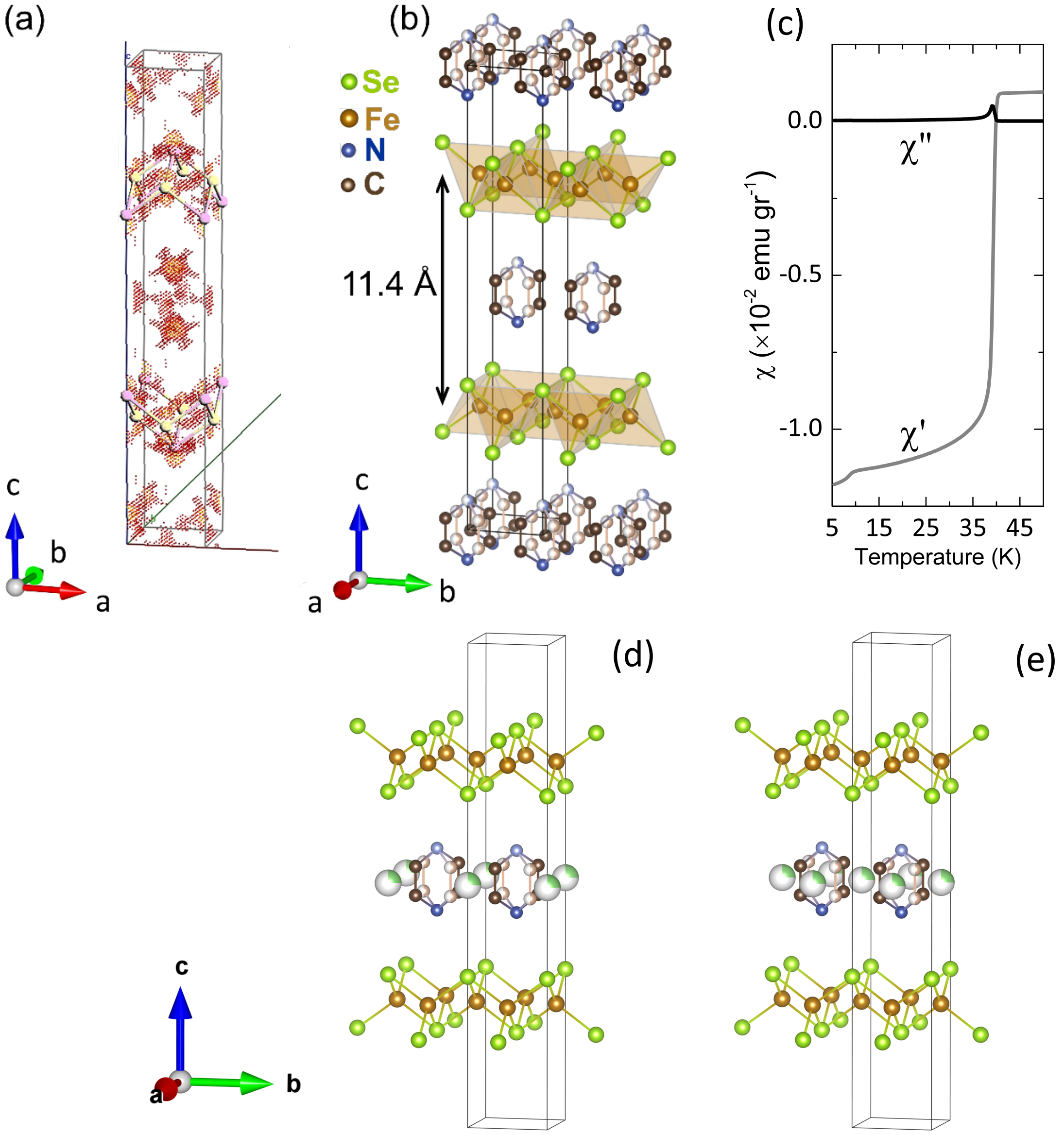

Figure S1. $Li_x(C_5H_5N)_yFe_{2-z}Se_2$: (a) Residual electron density clouds derived from difference Fourier map analysis in the TOPAS suite of programs. (b) A candidate crystallographic model, suggested from difference Fourier map analysis (i.e., $ThCr_2Si_2$, *122* structure-type, *I*4/*mmm*; *d*= 11.4 Å) of the high-T_c derivative, incorporating orientationally disordered pyridine molecules. (c) AC susceptibility (H_{ac}= 1 *Oe*, *f*= 999 Hz) of the sample batch measured by synchrotron XRD. Graphic depicting likely Li placement at sites, 2*b* (0, 0, ½) (d) or 4*c* (0, ½, 0) (e) of the $ThCr_2Si_2$ structure-type; Li sites were not part of the Rietveld refinement. Note: the model assuming the 2*b* site offers mean Li-N/C distances of about ~3 Å, but the 4*c* site is likely less favorable, as amongst the longer Li-N/C distances (~2.4 – 2.9 Å), the model predicts chemically less reasonable short Li-C bonds (~1.5 Å). The latter results in a relatively more distorted Li-Py coordination than the more symmetric one adopted for the 2*b* sites model.

S1.3 Rietveld analysis

Rietveld refinements optimized the structural configuration proposed by TOPAS. The suggested model was tested against both high-resolution (λ= 0.6193 Å) and lower-resolution data (**Figure 1b,c**) collected with a 2D detector. The multiphase Rietveld fits required $Li_x(C_5H_5N)_yFe_{2-z}Se_2$ as a majority phase, with β-FeSe and α-Fe as minority components; the sum of the scale factors for the three phases was constrained to be equal to one. Subsequent refinements corroborated that intercalated Py molecules were a constituent part of the compound. This was verified by having the N and C atoms (composing orientationally disordered Py molecules) incorporated at the 4*e* (4*mm*) and 16*m* (..*m*) sites, respectively, of the expanded lattice unit cell, in the *I*4/*mmm* space group.

For example, the Rietveld analysis of the 20 K XRD pattern, produced a relatively low residual (R_w= 14.4%, GOF= 2.1, N_{var}= 16; *I*4/*mmm*) when refinements included the N, C sites in the model. In the case where these sites were omitted from the crystallographic model, a higher residual (R_w= 22.0%, GOF= 3.2, N_{var}= 14) was obtained. In particular, the poorer fit in the absence of molecules, underestimates the (*004*), (*103*), and (*105*) Bragg peaks. The good quality-of-fit when C and N atoms are part of the intercalated phase's asymmetric unit supports the molecule's presence in the interlayer space.

As the N, C atoms are weak X-ray scatterers compared to the Fe, Se counterparts, a chemical constraint was utilized in order to facilitate the Rietveld refinements. For this, the site occupancies, η, of N and C atoms were linked to vary in a fixed ratio (η_N:η_C= 2) so that together with the corresponding atom site multiplicities, m, the disordered molecule's chemical composition is preserved. Rietveld refinements could then offer an upper estimate of the amount of molecules per unit cell when the total number of the molecule's constituent (N, C) atoms is evaluated. Here, the refined occupancies, obtained from medium-resolution XRD data and appended in **Table S1** (**Table S2**; high-resolution XRDs), suggest, $\eta_{\mathrm{N}} \times \mathrm{m_N} + \eta_{\mathrm{C}} \times m_{\mathrm{C}}$ = 0.95 × 4 + 0.475 × 16= 11.4 (or ~8.8) atoms per unit cell. Assuming that 6 (N,C) atoms are present in a molecule, it is proposed that ~1.9 (or ~1.4) molecules are contained per unit cell, equivalent to an upper estimate of y~0.9 (or ~0.7; high-resolution XRDs) for the intercalated molecule; with a lower bound of y esds ±0.1.

The model, with the Py molecules having an orientationally disordered configuration in between the close-to-stoichiometric Fe-Se sheets, after refinement suggests a composition of $Li_x(C_5H_5N)_yFe_2Se_2$ (x~ 0.6; y~ 0.7-0.9±0.1). The crystallographic parameters are compiled in the **Tables S1, S2**.

Table S1. Rietveld refined crystallographic parameters, at 300 K, of $Li_x(C_5H_5N)_yFe_2Se_2$ (x~ 0.6; y~ 0.9±0.1) (λ= 0.20735 Å; SDD: 2300 mm), and of β-FeSe (λ= 0.12203 Å; SDD: 1509 mm), from medium-resolution synchrotron XRD, measured with a 2D detector. The refinement involved the expanded-lattice $Li_x(C_5H_5N)_yFe_2Se_2$ as majority component (Phase I: *I4/mmm*), with β-FeSe (Phase II: *P4/nmm*) and α-Fe (Phase III: $Im\overline{3}m$) as secondary phases. The average refined weight percentages of these phases are: Phase I= 68.2(6) %, Phase II= 31.6(6) %, and Phase III= 2.4(5) %. For the parent phase the refinement involved β-FeSe as majority component (Phase I: *P4/nmm*), with α-Fe (Phase III: $Im\overline{3}m$; No. 229) as a minority phase. The average refined weight percentages of these phases, are: Phase I= 98.77(8) %, and Phase II= 1.22(8) %.

Sample	$Li_x(C_5H_5N)_yFe_2Se_2$	β-FeSe
Space group	*I4/mmm* (no. 139) [a, c]	*P4/nmm* (No. 129, choice 1) [b, c]
T_c (K)	40	8
$a = b$ (Å)	3.8424(3)	3.7688(1)
c (Å)	23.336(3)	5.5162(2)
V (Å^3)	344.54(7)	78.285(6)
occ (Se)	0.965(9)	0.994(4)
occ (N)	0.95(1)	-
occ (C)	0.475(5)	-
*z*Se	0.3129(1)	0.2665(2)
*z*N	0.436**	-
*z*C	0.470**	-
Fe-Se (Å) × 4	2.411(4)	2.3881(4)
Fe-Fe (Å) = $a/\sqrt{2}$	2.717(4)	2.6649(1)
$\mathrm{Se}-\widehat{\mathrm{Fe}}-\mathrm{Se}$ (α) (°) × 2	104.8(2)	103.99(3)
$\mathrm{Se}-\widehat{\mathrm{Fe}}-\mathrm{Se}$ (β) (°) × 4	111.81(2)	112.37(3)
Anion height, h_z (Å)	1.457(1)	1.467(1)
Volume $FeSe_4$ (Å^3)	7.146(1)	6.8524(1)
U_{iso} (Fe) (Å^2)	0.025(2)	0.0083(3)
U_{iso} (Se) (Å^2)	0.021(1)	0.0096(2)
U_{iso} (N) (Å^2)	0.01*	-
U_{iso} (C) (Å^2)	0.01*	-
R_w %	10.91	6.85
GOF	17.25	3.14
N_{obs}	1858	2625
N_{var}	17	10

Atomic sites:

[a] Fe 4*d* (0, ½,¼); Se 4*e* (0,0,*z*); N *4e* (½,½,*z*); and C 16*m* (0.72,0.72,*z*)

[b] Fe 2*a* (0,0,0); and Se 2*c* (0, ½,*z*)

[c] Fe-site occupancy was refined and found to be stoichiometric within the estimated standard error (esd); it was then fixed to 1.0.

* not refined

** derived from Fourier map analysis from TOPAS

S1.4 On the possible organization of Li-Py in the interlayer space

Difference Fourier map analysis via TOPAS (§S1.2) has suggested atomic sites with residual electron density near the Fe-Se sheets. This has been taken as relevant experimental evidence to infer the Py ring C_2 axis orientation with respect to the host layers (**Figure 1**), a model that has been refined by the Rietveld method against the synchrotron XRD data. Below a rationale is provided on the chosen configuration of Py and its likely coordination by Li that is consistent with the chemistry of polar aromatic molecules' that solvate polarizing electropositive cations.

It is well known that aromatic hydrocarbon molecules with sufficient electron affinity react with electropositive alkali metals in solutions to form principal ionic species that are monovalent anions. These ions result from the addition of a single electron to the lowest unoccupied π* antibonding orbital of the hydrocarbon and are actually radical anions.[3] Moreover, numerous investigations propose that Li atoms would interact with the aromatic π-system to coordinate above the aromatic plane and form complexes in both central and/or edge regions of the aromatic molecule.[4–6]

Along these principles, Py as an aromatic N-heterocyclic compound is also found to solvate alkali metals efficiently and its reactivity can be considered to occur in the afore-mentioned fashion, thus giving rise to [$C_5H_5N^{\cdot -}$] radical anions (R), with intensely colored solutions under air- and moisture- free conditions. Bearing in mind requirements for efficient charge transfer from Li atoms to the aromatic rings, one possible configuration that optimizes the electrostatic interaction among the [R – Li^+] species can assume that Li is located at the 2*b* site (0,0,½) or the 4*c* site (0,½,0) of the *I*4/*mmm* crystal lattice – sites also suggested for the isostructural [Li-NH_3] intercalated FeSe superconducting phases.[7] Then, a sublattice of atoms in the van der Waals gaps may be arranged so to coordinate either side of Li, forming layers of […R – Li^+ – R…], as depicted in **Figure S1d,e**.

Such a chemistry-imposed local geometry, due to the Li interaction with the aromatic π-system (absent in the NH_3-intercalated derivative), would prefer that the N orbital lone pair orientation is directed towards the Fe-Se sheets (i.e., with the pyridine C_2 axis perpendicular), allowing for optimal interaction of the lone pair with the layer electronic states. Having the aromatic N-heterocyclic accommodating additional electron density from Li, the N site would become more basic, and a possible configuration such as that shown in **Figure S1d** would promote electron transfer to the host sheets (cf., Lewis acid-base interaction scheme). Overall, this chemistry-imposed geometry for the interlayer species, with the nitrogen atoms located close to the host layers, may also instigate the change in the stacking sequence of the Fe-Se layers from *P* (primitive) in the parent β-FeSe to an *I* (body-centered) lattice upon intercalation.

S2. Total scattering

S2.1 Qualitative assessment

Our total scattering study shows a diffuse signal in the $F(Q)$ functions of both FeChs but with more pronounced features in the intercalated system (T= 300 K). A damped sine function, $y = y_0 + A \times e^{-\frac{x}{t_0}} sin\left(\pi \frac{x-x_0}{w}\right)$, has been used to fit the observed diffuse signal, in the range of $12 \leq Q \leq 22$ Å^{-1} (inset; **Figures 2a,b**) enabling the quantification of the amplitude (A) and frequency ($f = \frac{1}{2w}$) of these oscillations. The fitted parameters for the $Li_x(C_5H_5N)_yFe_{2-z}Se_2$ yield, $A = 5.2(4)$ Å^{-1}, with w= 1.322(3), while for the parent β-FeSe yield, $A = 1.3(2)$ Å^{-1}, and w= 1.362(6). The diffuse signal indicates an increased disorder when one goes from the parent to the $Li_x(C_5H_5N)_yFe_{2-z}Se_2$ derivative.

The effects in turn, reflect in the evolution of the $G(r)$ function (**Figure 2c,d**). The more pronounced disorder in $Li_x(C_5H_5N)_yFe_2Se_2$ makes the first PDF peak (cf., Fe-Se bonding, at r=2πf) to appear sharper compared to higher-r peaks, which are damped and broadened, signifying weaker atomic correlations. The latter suggest that far-neighbor atom-pairs after intercalation do not move in phase, indicating a structure with short-range incoherent local domains.

S2.2 Quantitative model-based assessment.

Aiming to evaluate further the above findings, model-dependent fitting of the 300 K $G(r)$s (Q_{min}= 0.1 Å^{-1}, Q_{max}= 21.9 Å^{-1}, r_{poly}= 0.9) was carried out. The $G(r)$ data from the calibrants were refined to obtain instrument-resolution parameters, namely: $Q_{damp,Ni}$= 0.07660(1) Å^{-1} (λ = 0.12203 Å; SDD: 357 mm), and $Q_{damp,LaB6}$= 0.044(1) Å^{-1} (λ= 0.20735 Å; SDD: 280 mm).

The PDF fit of the parent β-FeSe material initially was carried out with the tetragonal model (*P*4/*nmm*) suggested by the Rietveld analysis, which also included the cubic α-Fe as a secondary phase (**Table S1**). This supports the tetragonal symmetry as a good descriptor of the average structure (r= 2-40 Å; R_w= 7.03%; **Figure S2a**). However, a $G(r)$ feature in the local structure ($r\sim$ 3.6-3.7 Å) is inadequately described. This involves NN Se-Se pair distances and may indicate possible electronic nematic fluctuations, according to earlier reports which have suggested room-temperature local nematicity in β-FeSe superconductor.[8] Utilizing this approach for the present PDF analysis, a local orthorhombic distortion was assumed that required the *Cmma* symmetry (**Figure 2d**). This model provides a somewhat better description of the NN Se pair correlations (cf., $r\sim$ 3.6-3.7 Å) as compared to the tetragonal model (**Figure S2b, c)**. Comparison of relevant structural parameters derived from PDF analyses are compiled in the Table accompanying **Figure S2**.

A similar procedure was attempted for the analysis of the $G(r)$ data for $Li_x(C_5H_5N)_yFe_{2-z}Se_2$. The tetragonal model (*I*4/*mmm*) suggested by Rietveld analysis (**Table S1**) was utilized as a starting point for the PDF refinement in the region of higher, r= 2-40 Å; the model incorporated also low-content secondary phases, as the parent β-FeSe and α-Fe. The resulting PDF fit (**Figure S3a**; R_w= 19.4%) indicates that the average structure

model does not describe well this intermediate r-range $G(r)$ data, likely reflecting significant structural disorder that is not adequately captured by the PDF model. This disorder, however, is sufficiently accounted for in the Rietveld refinements through the inclusion of microstrain broadening along different crystallographic directions (see §S3.2 below and §3.2.1). The discrepancy can be rationalized since the Rietveld captures average long-range order, whereas PDF analysis is sensitive to various length scales, including local.

To further explore the influence of disorder at the nanoscale, PDF refinements in the region of lower, r= 2-10.5 Å, were performed. As starting point for the $Li_x(C_5H_5N)_yFe_2Se_2$ phase was taken again the tetragonal model ($I4/mmm$) suggested by the Rietveld analysis (**Table S1**), including the low-content secondary phases. Interestingly, the resulting PDF fit (**Figure S3c**; R_w= 11.8%) indicates that a tetragonal local structure is a sufficient description of the nanoscale $G(r)$ region. Structural parameters derived from PDF analysis are compiled in the Table accompanying **Figure S3**. The validity of the tetragonal local structure model over longer length scales was also assessed on the basis of "box-car" PDF fits.[8] "Box-car" type of fitting was performed with 15 different 8.5 Å broad r-windows by shifting the window-centroid (r_m) in steps of 0.8 Å from 6.5 Å to 18 Å. The evolution of the quality-of-fit factors (R_w) from the ''box-car'' PDF fittings are shown in **Figure S3b**, while some representative fits in **Figure S3d-f**. Each distinct r-range fit provides structural information relevant to a corresponding radial distance window, allowing for quantification of the variation of local structure as a function of r (i.e., the correlation length of local features). Such analysis confirms the increased disorder in the $Li_x(C_5H_5N)_yFe_2Se_2$ by demonstrating that the tetragonal model fits well the nanoscale region (r< 10.5 Å), but progressively becomes insufficient as one moves to incorporate the higher r-region (**Figures S3**). The steeper rise of R_w at about ~1.2 nm suggests that the characteristic size of possible incoherent local domains may be comparable.

A possible deviation from tetragonality[8] at the nanoscale has also been considered. The lower-r PDF analysis though rules out such a distortion under the present experimental conditions (see §S2.3 below).

Figure S2. *P4/nmm vs. Cmma* PDF analysis of β-FeSe

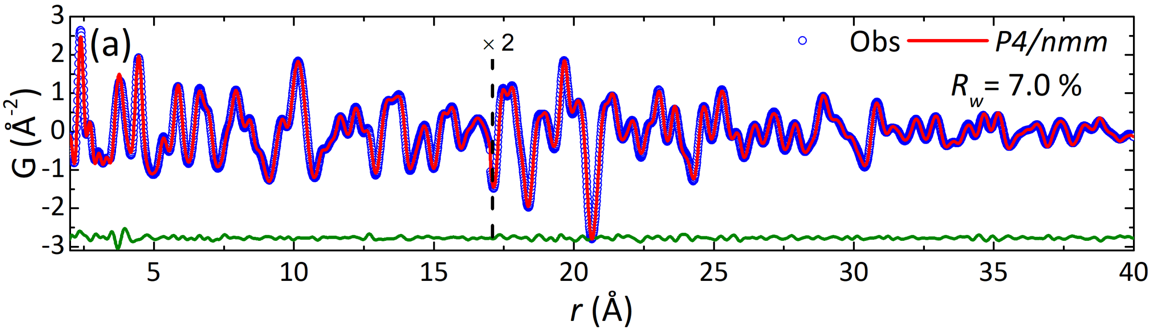

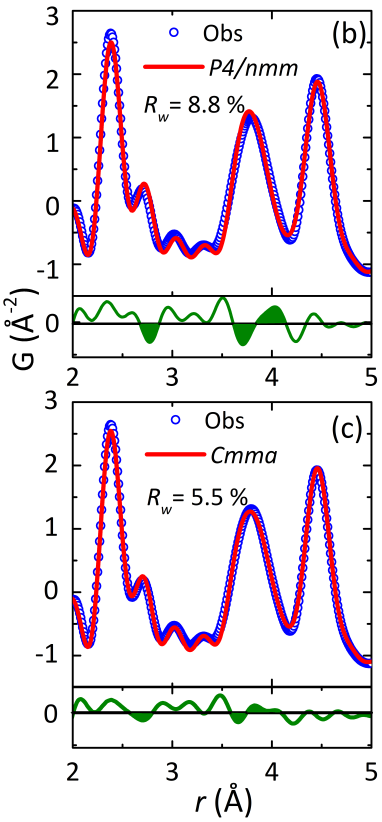

Parameter	***P4/nmm***		***Cmma***
R-range (Å)	2-40	2-5	2-5
R_w (%)	7.03	8.78	5.50
a (Å)	3.766	3.782(4)	5.367(4)
b (Å)			5.323(3)
c (Å)	5.511	5.46(1)	5.500(2)
r(Fe-Se) (Å) × 4	2.3879	2.3868(6)	2.3884(1)
r(Fe-Fe) (Å)	2.663	2.674(3)	2.661(2)
			2.683(2)
r(Se-Se) (Å)	3.704	3.695(2)	3.706(3)
	3.766	3.782(4)	3.722(4)
			3.779(2)
	3.964	3.954(3)	3.952(3)
			3.967(3)
$Se - \widehat{Fe} - Se$ (α) (°)	104.11 × 2	104.19(1) × 2	104.59(1) × 2
$Se - \widehat{Fe} - Se$ (β) (°)	112.22 × 4	112.18(1) × 4	111.65(1) × 2
$Se - \widehat{Fe} - Se$ (γ) (°)			112.28(1) × 2
Anion height, h_z (Å)	1.468	1.467(3)	1.471(3)
Volume $FeSe_4$ (Å^3)	3.470	3.469(1)	3.497(5)

Figure S2. PDF analysis of the *G*(*r*) data at 300 K for β-FeSe. Refinements: (a) extended to the higher, *r*= 2-40 Å, with the tetragonal *P4/nmm* model, and with focus in the nanoscale region, *r*= 2-5 Å, adopting the tetragonal *P4/nmm* (b) and the orthorhombic *Cmma* (c) models. *G*(*r*) observed data (blue points), small-box model (red lines), and their difference (green traces). The table compares the derived structural parameters, obtained from the PDF analysis, using either the tetragonal or orthorhombic models for the nanoscale and longer-length scale (*r*= 2-40 Å) regions.

Figure S3. 'Box-car' PDF assessments of $Li_x(C_5H_5N)_yFe_2Se_2$

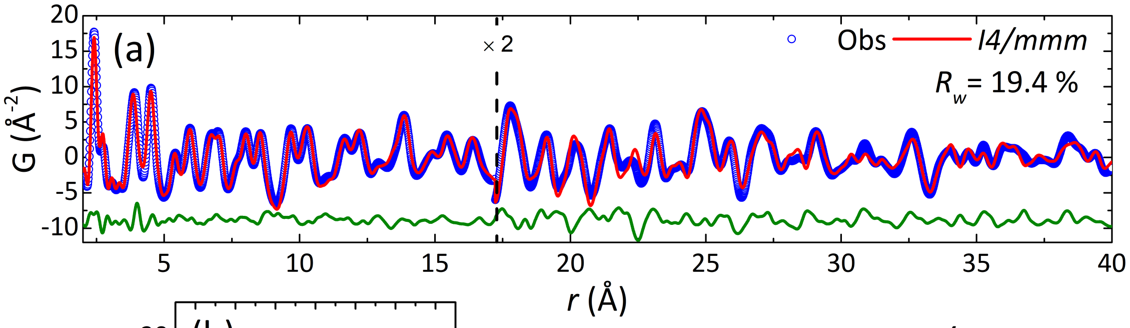

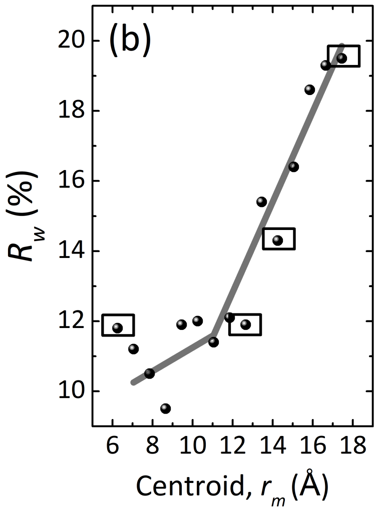

Parameter	**I4/mmm**
r-range (Å)	2-10.5
R_w (%)	11.80
a, b (Å)	3.8382(4)
c (Å)	23.41(1)
r(Fe-Se) (Å) × 4	2.4162(4)
r(Fe-Fe) (Å)	2.7163(3)
r(Se-Se) (Å)	3.8388(5)
	3.998(1)
$Se-\widehat{Fe}-Se$ (α) (°) × 2	105.18(3)
$Se-\widehat{Fe}-Se$ (β) (°) ×4	111.63(2)
Anion height, h_z (Å)	1.465(1)

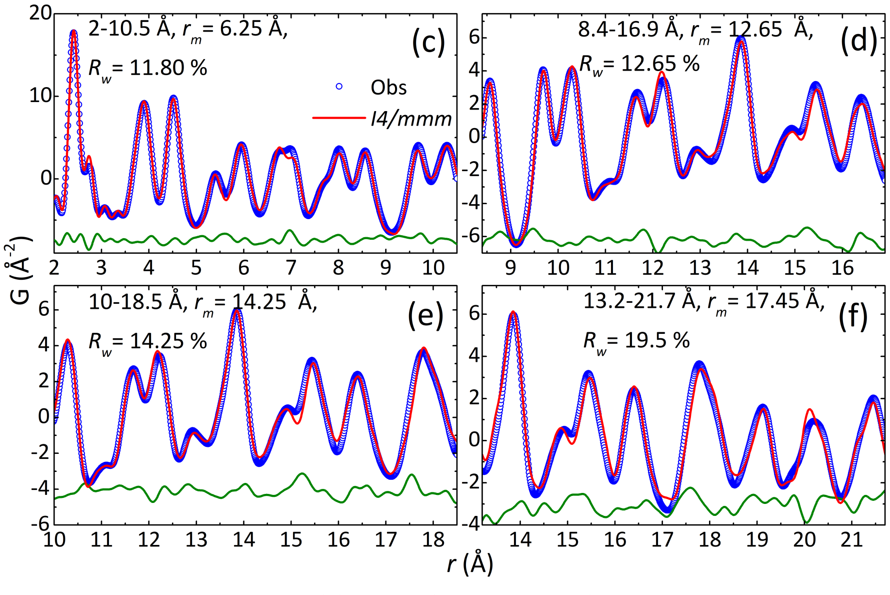

Figure S3. PDF analysis of the $G(r)$ data (blue points) at 300 K with the tetragonal *I4/mmm* model (red lines) of the $Li_x(C_5H_5N)_yFe_2Se_2$ as a majority phase; difference curve (green traces). (a) PDF refinement in the region of higher, *r*= 2-40 Å. (b) Evolution of the quality-of-fit factors (R_w) obtained from "box-car" PDF refinements in the region, *r*= 2-25 Å. The black spheres represent the centroids of each narrower *r*-window. Points marked with 'squares' identify the PDF fits that are presented in panels c-f. (c) The initial *r*-window, $2 \leq r \leq 10.5$ Å, (d) the next window, spans from $8.4 \leq r \leq 16.9$ Å, while (e) covers $10 \leq r \leq 18.5$ Å , (f) final *r*-window, from $13.2 \leq r \leq 21.7$ Å. The corresponding quality-of-fit factors, R_w (%), are provided.

S2.3 Local orthorhombicity in $Li_x(C_5H_5N)_yFe_2Se_2$.

Earlier reports of electronic, local nematicity that imposes a symmetry-breaking, orthorhombic distortion (*Cmma*) in β-FeSe at room temperature, require additional PDF analysis for the intercalated to verify or rule-out a similar tetragonal to orthorhombic distortion with possible short-range correlations. To test if a symmetry-lowering structural response manifests at the local scale in the intercalated, the model should be corroborated by changes in the atom-atom pair correlations, (i) encompassing the stacking direction of the Fe-Se sheets (e.g., nearest interlayer distances are, Se-Se~ 8.5 Å) and/or (ii) within the thickness of the Fe-Se sheets themselves.

Therefore, $Li_x(C_5H_5N)_yFe_2Se_2$ 300 K PDF refinements began with the intermediate *r*-range, $2\leq r\leq 9$ Å, which primarily captures extended, interlayer lattice correlations and then focused on the very short-range, $2\leq r\leq 5$ Å, where orthorhombic distortions may be prominent, as observed in the parent β-FeSe. Specifically: (i) Interlayer pair-correlations ($2\leq r\leq 9$ Å): A direct comparison of the PDF fits using the tetragonal and orthorhombic models reveals that the orthorhombic yields a higher R_w (12%; **Figure S4a**) relative to the tetragonal model (10.8%; **Figure S4b**). The reduced fit quality is particularly evident at *r*~ 3.6-3.7 Å by a subtle misfit, which corresponds to the NN Se–Se pair distances peak. Additional misfits appear in the *r*~ 8.5 Å, where the interlayer Se-Se distances reside. (ii) Intralayer pair-correlations ($2\leq r\leq 5$ Å): the *I*4/*mmm* setting describes well the *G*(*r*) of the intercalated relative to the *Cmma* which exhibits a misfit around *r*~ 3.6 Å (**Figure S4c,d**; see also difference plots). The *Cmma* though, provides a statistically improved description of the *G*(*r*) for β-FeSe (**Figure S4e,f** and §S2.2).

The orthorhombic model attempts to broaden the feature at *r*~ 3.6 Å by introducing increased number of inequivalent Se–Se distances, as it would be expected for a locally broken symmetry, cf., β-FeSe. As the experimental *G*(*r*) data do not show unambiguous evidence for such peak broadening, the tetragonal local structure model, with the least number of variables, is favored for the intercalated phase. Collectively, the PDF analysis suggests that a tetragonal to orthorhombic distortion, with short-range correlations (between distortion directions of different layers) due to a similar nature, electronic nematic fluctuations, such as those met in β-FeSe,[8] is not evident under the present experimental conditions in the intercalated system.

Figure S4. *I4/mmm vs. Cmma* PDF analysis of $Li_x(C_5H_5N)_yFe_2Se_2$

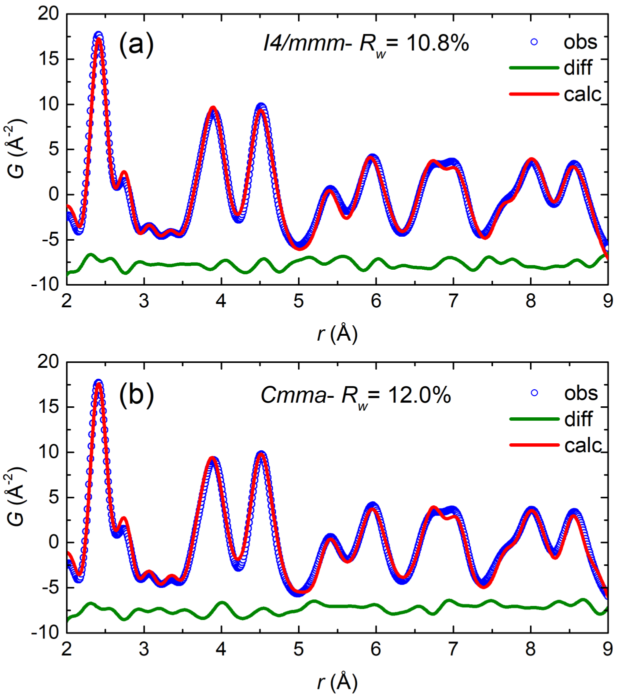

Parameter	***I4/mmm***	***Cmma***
r-range (Å)	2-9	2-9
R_w (%)	10.8	12.0
a (Å)	3.842(2)	5.399(7)
b (Å)		$a_T \times \sqrt{2}$= 5.438
c (Å)	23.2	11.6
r(Fe-Se) (Å) × 4	2.415(1)	2.416(2)
r(Fe-Fe) (Å)	2.717(1)	2.696(5)
		2.718
r(Se-Se) (Å)	3.842(2)	3.829(3)
	3.994(1)	3.994(5)
		4.009(2)
$Se-\widehat{Fe}-Se$ (α) (°)	105.38(4) × 2	104.83(3) × 2
$Se-\widehat{Fe}-Se$ (β) (°)	111.55(2) × 4	111.51(7) × 2
$Se-\widehat{Fe}-Se$ (γ) (°)		112.16(8) × 2
Anion height, h_z (Å)	1.463(2)	1.463(5)

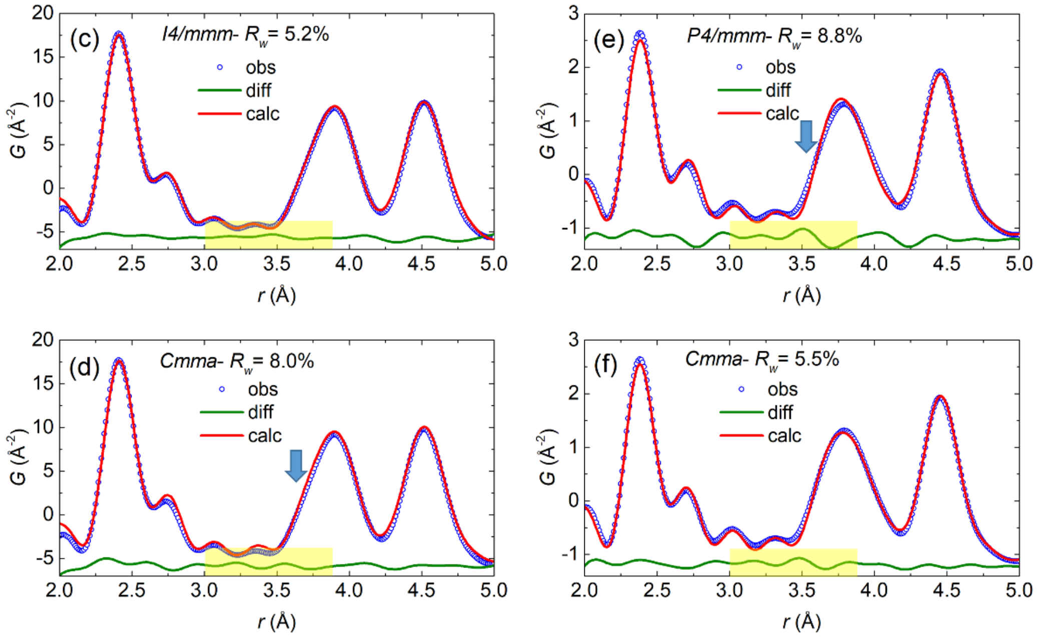

Figure S4. PDF refinements of the 300 K *G*(*r*) data (blue points) for $Li_x(C_5H_5N)_yFe_2Se_2$ main phase. *r*= 2–9 Å, compares the tetragonal *I4/mmm* (a), with the orthorhombic *Cmma* (b), crystallographic models (red lines); difference curve (green traces). *r*= 2–5 Å, with focus in the nanoscale region, compares (c) the tetragonal *I4/mmm* and (d) the orthorhombic *Cmma* models. Arrow marks the misfit at *r*~ 3.6 Å of the *Cmma* (see difference). PDF fits of the reference β-FeSe at 300 K are shown using (e) the tetragonal *P4/nmm* and (f) the orthorhombic *Cmma* crystal lattice settings. Arrow marks the misfit at *r*~ 3.6 Å in the *P4/nmm* (see Figure S2b,c).

S.3 Temperature-dependent Rietveld analysis

S3.1 Quest for symmetry-lowering global distortion

In iron superconductors electronic nematicity is commonly manifested as an in-plane distortion of the Fe-pnictogen or Fe-chalcogen plane from a tetragonal (*a=b*, *c*) to orthorhombic (*a≠b*, *c*) lattice. So, searching for evidence that support a symmetry-lowering, orthorhombic distortion necessitates investigating the diffraction data through basal plane lattice descriptions where the C_4 symmetry is no more preserved and as such, a symmetry-breaking electronic phase might emerge as a viable driver.

The rational of section S3.1 rests on the above concept aiming to disregard that the NTE is attributable to an obscure global symmetry-lowering distortion. So, initially we discuss the structure evolution upon cooling the $Li_x(C_5H_5N)_yFe_2Se_2$ (x~ 0.6; y~ 0.7±0.1) sample down from 150 to 20 K. High-resolution XRD powder patterns at T≤ 150 K display peak-shape variations and broadening (also evaluated in subsequent Rietveld refinements; §S3.2) at high-*Q*. In assessments with the tetragonal *I*4/*mmm* model as a majority $Li_x(C_5H_5N)_yFe_2Se_2$ phase, the apparent increase in the number of predicted Bragg peaks in the high *Q*-region (3.40-3.50 Å), is attributed to *T*-induced lattice-size changes (**Figure S5a-c**). Upon cooling, previously overlapping peaks at high-*Q* became better resolved, with a modest evolution of R_w (**Figure S5d**), importantly though, patterns remained well-indexed on the basis of the *I*4/*mmm* symmetry. From this evidence alone, one can conclude that symmetry-lowering, as could be substantiated by the emergence of new Bragg reflections visibly resolved in the powder patterns, is not in place (i.e., absence of any orthorhombic splitting of the Bragg reflections). The chosen tetragonal model describes well the average structure of the intercalated phase both above and below T_S (~70 K) where NTE is witnessed to emerge.

Beyond this basic evaluation, as electronic nematicity imposes a global symmetry-breaking (T_{nem} < 90 K) in the parent β-FeSe, we considered that a simple quantitative test of the $Li_x(C_5H_5N)_yFe_2Se_2$ XRD data against the known orthorhombic distortion (*Cmma*) of the parent may provide extra confidence on the inadequacy of the specific electronic process when extrapolated to the intercalated phase. Consequently, this was not meant be an exhaustive test utilizing different orthorhombic space groups, as the *I*4/*mmm* symmetry already indexes well the XRD data for the intercalated. Nevertheless, a comparison of the Le Bail refinements using the tetragonal (*I*4/*mmm*; R_w= 14.45%) and orthorhombic (*Cmma*, with a $\sqrt{2}\times\sqrt{2}$ supercell enlargement for the basal plane;[9] R_w= 14.40%) models, shows that the weighted profile *R*-factors (R_w) are comparable (**Figure S6**). Given that the tetragonal model reproduces the observed diffraction pattern with fewer variables (N_{var}), it is the preferred choice, indicating that the *I*4/*mmm* space group offers an optimal description of the structure after intercalation (**Figure 1a**). The present analysis suggests no global symmetry-lowering.

Figure S5. T-dependent Rietveld, high-*Q* features

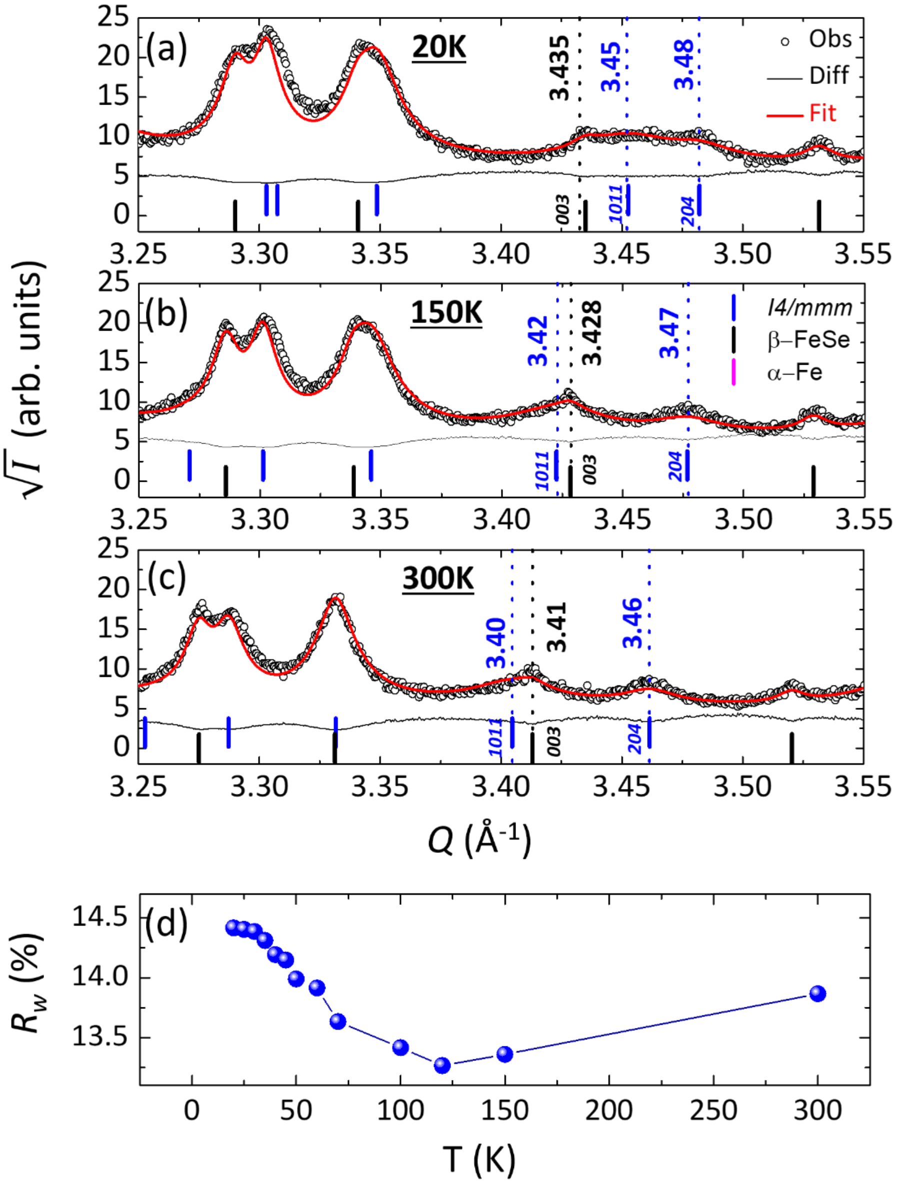

Figure S5. Portion of the high-*Q* region, from Rietveld refinements displaying typical evolution of the patterns upon cooling (a, b, c). The Stephens phenomenological model of anisotropic peak broadening was utilized. Tick marks: purple, correspond to $Li_x(C_5H_5N)_yFe_2Se_2$ (x~ 0.6; y~ 0.7±0.1), in *I*4/*mmm* symmetry and black to the parent β-FeSe, in *P*4/*nmm* symmetry. (d) the temperature evolution of the quality-of-fit factors, R_w, upon cooling. Modeling without the Stephens' anisotropic peak broadening, results in significant deterioration of R_w – refer to Figure S6 below.

Figure S6. Test for global symmetry-lowering in the intercalated lattice

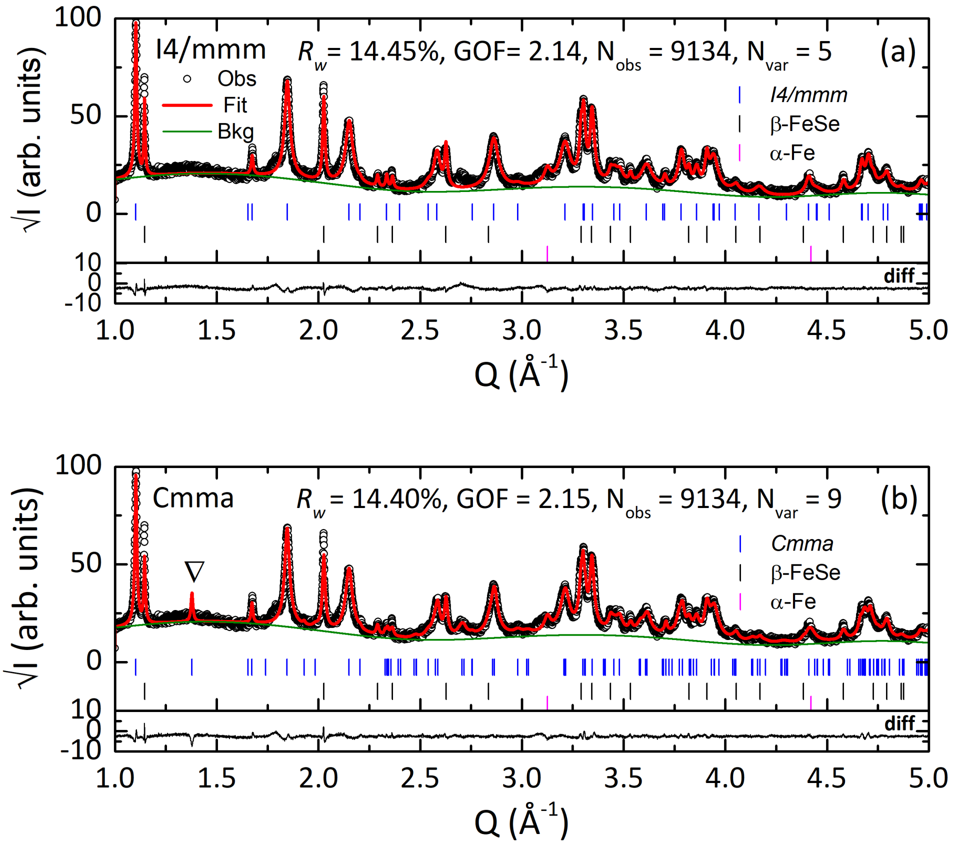

Figure S6. Le Bail full profile analysis (λ= 0.6193 Å) of the $Li_x(C_5H_5N)_yFe_2Se_2$ at 20 K, comparing the higher-*Q* region for (a) the tetragonal [*I4/mmm*; *a*=*b*= 3.8044(1) Å, *c*= 22.7945(4) Å; refer to Figure 1a for full *Q*-coverage] and (b) orthorhombic [*Cmma*; *a*= 5.3735(4) Å, *b*= 5.3885(4) Å, *c*= 22.7949(4) Å] lattice models. The intercalated lattice was the majority phase, with β-FeSe and *α*-Fe, as minority components. (∇) systematically absent reflection.

S3.2 Anisotropic microstrain peak broadening

The temperature-dependent variations of the Bragg reflections in the Rietveld refined patterns at high-Q inquire about microstrain-induced peak broadening in the high-resolution XRD data of $Li_x(C_5H_5N)_yFe_2Se_2$ (x~ 0.6; y~ 0.7±0.1).

In the general case the spacing, d between lattice planes for any given reflection defined by the Miller indices, *hkl* is given by,

$$1/d^2 = M_{hkl} = Ah^2 + Bk^2 + Cl^2 + Dkl + Ehl + Fhk \quad (1)$$

where {A; . . . ; F} are metric parameters of the reciprocal lattice. Strain broadening then is considered as a manifestation of the distribution of such parameters. The GSAS suite[1] notation for strain that leads to anisotropic line broadening calculates,

$$\Delta d/d = \pi[\sigma^2(M_{hkl})]^{1/2}/(18000M_{hkl}). \quad (2)$$

The anisotropic contribution to the line broadening due to microstrain is parametrized on the basis of the generalized Stephens model (cf., S_{HKL} coefficients),[10]

$$\sigma^2(M_{hkl}) = \sum_{HKL} S_{HKL} h^H k^K l^L \quad (3)$$

with terms S_{HKL} defined for H+ K+ L = 4. The general (triclinic) case employs 15 terms that are reduced to only 4 for the tetragonal (*4/mmm*) symmetry,

$$\sigma^2(M_{hkl}) = S_{400}(h^4 + k^4) + S_{004}l^4 + 3S_{220}h^2k^2 + 3S_{202}(h^2l^2 + k^2l^2) \quad (4)$$

where, S_{400}=S_{040} and S_{202}=S_{022}.

On the above grounds, temperature-dependent Rietveld analysis of the $Li_x(C_5H_5N)_yFe_{2-z}Se_2$ indicates that implementation of the phenomenological microstrain model proposed by Stephens improves the quality-of-fit by reducing the value of residual (R_w) by ~4% (**Figure S7**). This is in marked contrast to the Bragg peaks in the patterns of the parent β-FeSe which are treated with the isotropic microstrain since no peak width variations are detected in the high-Q region.

With the extracted S_{hkl} coefficients, GSAS-II calculates the 3D isosurface for the representation of the distribution of microstrain (μstrain, $\Delta d/d \times 10^{-6}$) in the crystalline lattice. The variable distance of the 3D isosurface from the origin and its anisotropic morphology characterize the magnitude of the microstrain along different crystallographic directions. The 3D isosurfaces, at the two representative temperatures above and below T_c (**Figure S8**) are of similar truncated ellipsoidal geometry, showing a morphology with elongated x-y and squashed x-z, y-z cross sections. The shape informs that (i) the largest microstrain is within the x-y cross sections that correspond to in-plane lattice directions (cf., relevant to the Fe-square plane NTE effect; see §3.2.1), while (ii) it is somewhat lower within the out-of-plane x-z, y-z cross sections. In addition, there is no evidence for additional anisotropy in the x-y plane that could support the onset of an orthorhombic distortion (e.g., relevant to orthorhombic splitting of the Bragg reflections) upon cooling. The refinements provide the average distance from the origin, that is the mean microstrain value, which does not significantly change upon cooling through T_c, namely, μstrain-20 K = 20881 $\times 10^{-6}$, μstrain-150 K = 22543 $\times 10^{-6}$.

Figure S7. Isotropic *vs.* anisotropic microstrain broadening.

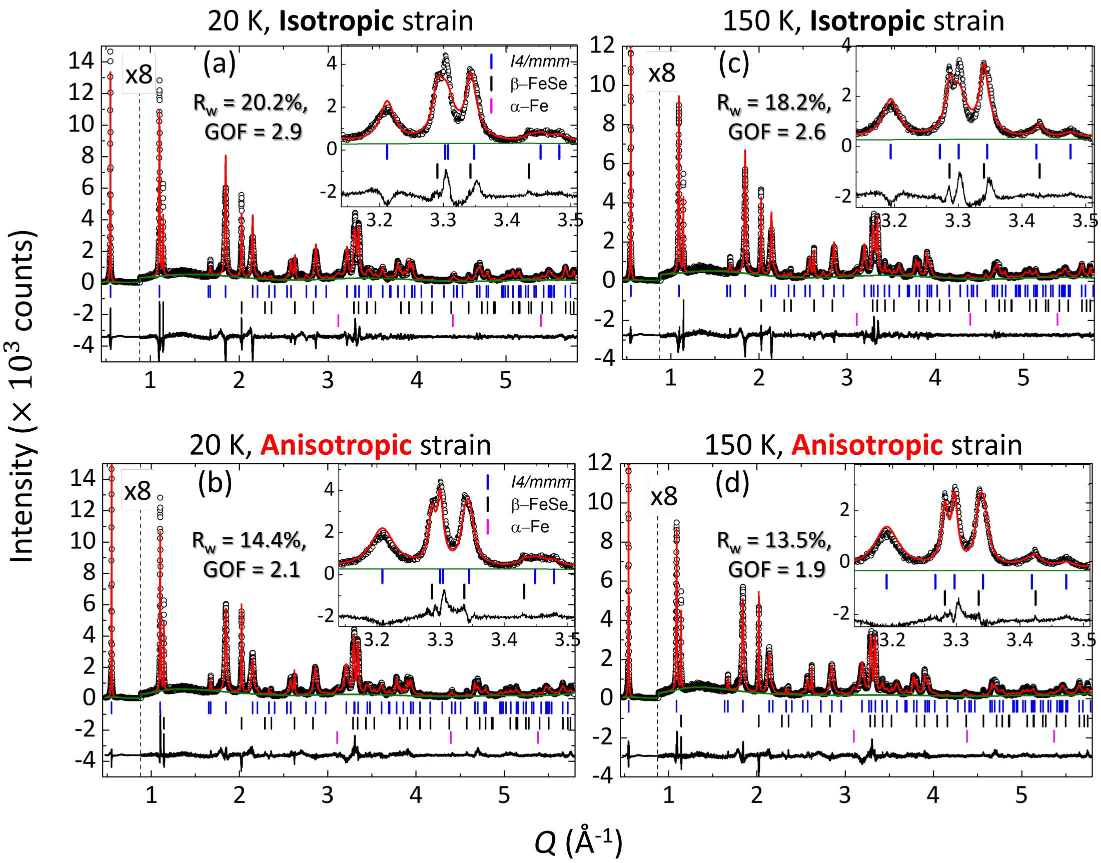

Figure S7. Typical Rietveld refinements (λ = 0.6193 Å) of $Li_x(C_5H_5N)_yFe_2Se_2$ (x~ 0.6; y~ 0.7±0.1) as a majority phase, with β-FeSe and α-Fe, as minority components. (a), (c): treating the majority phase with an isotropic strain model. (b), (d): utilizing the Stephens phenomenological model of anisotropic peak broadening to characterize the evolution of microstrain across the temperatures. The quality-of-fit factors (R_w, GOF) are included. The black points and red lines represent the data and calculated profile, respectively. The black line at the bottom is the difference between observed and calculated patterns. Tick marks depict the position of Bragg peaks for: (blue; *I*4/*mmm*) the intercalated lattice $Li_x(C_5H_5N)_yFe_2Se_2$, (black; *P*4/*nmm*) the parent β-FeSe, (magenta; $Im\bar{3}m$) cubic phase of the α-Fe.

Figure S8. Anisotropic microstrain

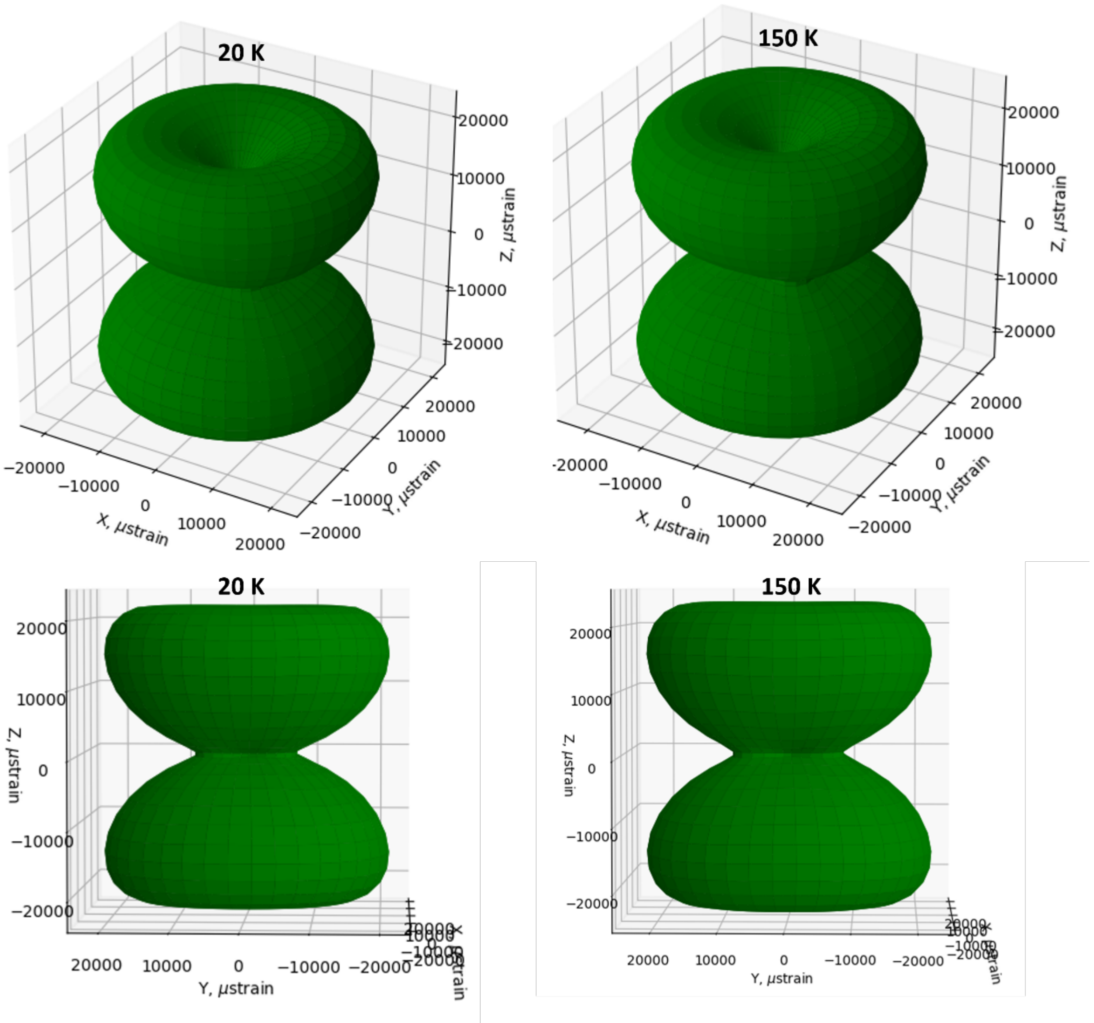

Figure S8. Three dimensional isosurface of the experimentally determined microstrain (µstrain, Δ*d*/*d* ×10^{-6}) of the tetragonal $Li_x(C_5H_5N)_yFe_2Se_2$ phase.

Table S2. Rietveld refined crystallographic parameters of $Li_x(C_5H_5N)_yFe_2Se_2$ (x~ 0.6; y~ 0.7±0.1) from high-resolution synchrotron XRDs (λ = 0.6193 Å), at 20 K and 300 K. The refinements involved the expanded-lattice $Li_x(C_5H_5N)_yFe_2Se_2$ as majority component (Phase I: *I*4/*mmm*), with β-FeSe (Phase II: *P*4/*nmm*; No. 129, choice 1) and α-Fe (Phase III: $Im\overline{3}m$; No. 229) as secondary phases. The average refined weight percentages of these phases, at 20 K, are phase I: 60.4(3) %, phase II: 33.2(2)%, and phase III: 6.4(1)%.

Sample	**$Li_x(C_5H_5N)_yFe_2Se_2$**	
Space group	*I4/mmm* [a, c] (no. 139)	
T_c (K)	40	
Temperature (K)	20	300
$a = b$ (Å)	3.8088(1)	3.8269(1)
c (Å)	22.8209(4)	23.2066(5)
V (Å^3)	331.05(2)	339.86(2)
occ (Se)	0.994(1)	0.994(1)
occ (N)	0.728(5)	0.728*
occ (C)	0.364(2)	0.364*
zSe	0.3134(1)	0.31260(9)
zN	0.436**	0.436**
zC	0.470**	0.470**
Fe-Se (Å) × 4	2.392(1)	2.402(1)
Fe-Fe (Å) = $a/\sqrt{2}$	2.693(1)	2.706(1)
$\mathrm{Se}-\widehat{\mathrm{Fe}}-\mathrm{Se}$ (α) (°) × 2	105.51(8)	105.59(7)
$\mathrm{Se}-\widehat{\mathrm{Fe}}-\mathrm{Se}$ (β) (°) × 4	111.18(4)	111.95(4)
Anion height h_z (Å)	1.448(2)	1.452(2)
Volume $FeSe_4$ (Å^3)	6.746(1)	7.099(1)
U_{iso} (Fe) (Å^2)	0.0095(8)	0.0113(8)
U_{iso} (Se) (Å^2)	0.0072(5)	0.0109(5)
U_{iso} (N) (Å^2)	0.01*	0.01*
U_{iso} (C) (Å^2)	0.01*	0.01*
R_w %	14.4	13.9
GOF	2.1	1.7
N_{obs}	10168	10168
N_{var}	17	16

Atomic sites:

[a] Fe 4*d* (0, ½,¼); Se 4*e* (0,0,*z*); N 4*e* (½,½,*z*); and C 16*m* (0.72,0.72,*z*)

[b] Fe-site occupancy was refined and found to be stoichiometric within the esd; it was then fixed to 1.0.

* not refined

** derived from Fourier map analysis from TOPAS

S3.3 Thermal-expansion coefficients

The thermal expansion coefficients (TECs; **Figure 3b**) of a crystal lattice provide useful insights when correlation-driven instabilities emerge. The linear TECs α_{α}, α_{c}, for the *a,b*- and *c*-axis, respectively, are determined by calculating the temperature derivatives of the lattice parameters, as:

$$\alpha_{a,c} = \frac{1}{\alpha_{a,c\,(300\,K)}} \frac{\partial \Delta a,c}{\partial T}. \tag{5}$$

While the volume TEC *β* is calculated by taking the temperature derivatives of unit cell volume, as:

$$\beta = \frac{1}{V_{300\,K}} \frac{\partial \Delta V}{\partial T}. \tag{6}$$

S3.4 Geometrical parameters

Rietveld analysis from the high-resolution XRD data (T< 150 K) provides further information on the temperature evolution of the geometrical parameters that describe the $FeSe_4$ tetrahedral building blocks within the FeSe layers (**Figure 2e,f**). The intercalated $Li_x(C_5H_5N)_yFe_2Se_2$ displays longer Fe-Se bonds **(Figure S9a)** and increased Fe_4Se square pyramidal unit volume **(Figure S9b)** with respect to the parent β-FeSe. Additionally, the $\mathrm{Se}-\widehat{\mathrm{Fe}}-\mathrm{Se}$ bond angle (α) widens upon intercalation and annealing **(Figure S9c)**, while the anion-height (i.e., Se to Fe-sheet *c*-axis normal distance, h_z) values become shorter **(Figure S9d)**. In the high-T_c system, widening of the bond angle and compression of h_z suggest that the $FeSe_4$ units (**Figure 2e,f**) become squeezed along the *c*-axis, with concomitant shrinking upon cooling, when compared to the parent β-FeSe.

Importantly, as in other molecule-intercalated FeChs [cf., $Li_x(NH_2)_y(NH_3)_{1-y}FeSe$],[7] after Py intercalation, the anion height (h_z= 1.452(2) Å) and the bond angle ($\alpha\sim$ 105.59(7)°), remain away from what is empirically thought to be optimal (i.e., h_z= 1.38 Å and $\alpha\sim$ 109.47°) for high-T_c materials.[11] The subtle responses of θ(T) (**Figure 3b**) and S_{hkl}(T) (**Figure 4**) at T_c, where the local Fe-Se layer appears to be getting thinner (cf., h_z compressed; **Table 1**; **Figure S9b,d**) communicate a mutual relation to superconductivity, but still from the average structure point of view.

Figure S9. T-evolution of geometrical parameters

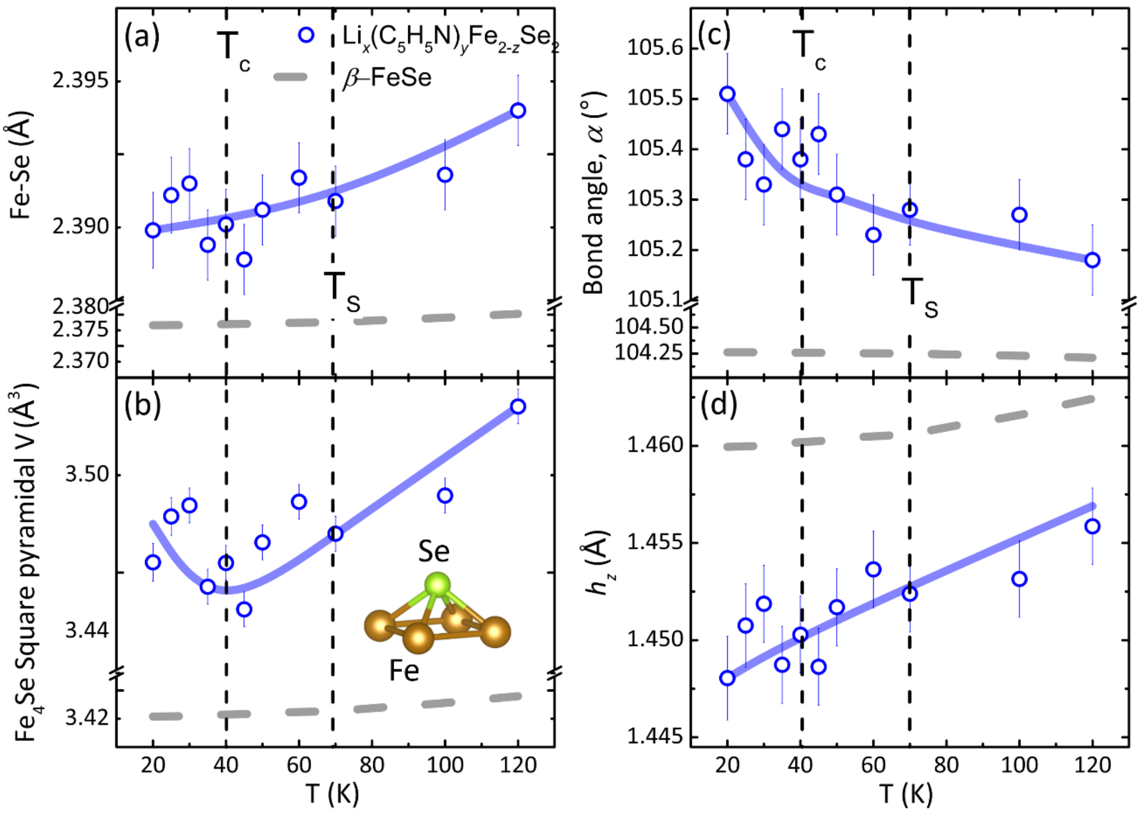

Figure S9. Temperature evolution of the (a) Fe-Se bond length, (b) volume of Fe_4Se square pyramidal building blocks, (c) $Se-\widehat{Fe}-Se$ bond angle, α and (d) anion height, h_z, derived after Rietveld refinements of the high-resolution synchrotron XRD patterns of the $Li_x(C_5H_5N)_yFe_2Se_2$ (x~ 0.6; y~ 0.7±0.1) (blue points; λ= 0.6193 Å) samples. Thick grey dashed lines, is a guide to the eye, portraying relevant structure metrics in β-FeSe[12]. The vertical dashed lines indicate the T_c= 39 K and the T_S= 70 K (cf., marking the onset of negative thermal expansion) for the $Li_x(C_5H_5N)_yFe_2Se_2$ sample.

S4. Core-level spectroscopy

Core level spectroscopy data were collected on highly crystalline, phase-pure the $Li_x(C_5H_5N)_yFe_2Se_2$ powder sample with superconducting critical temperature of T_c = 39 K (**Figure S10**).

S4.1 X-Ray Absorption Spectroscopy: normalization and modelling

The measured raw Fe-K edge XAS spectra have been normalized within the ATHENA software.[13] First, the threshold energy E_0, approximately in the middle of the absorption jump, was located by taking the maximum of the derivative in the absorption spectra, $\mu(E)$. Then, a smooth straight line was fitted to the pre-edge region to remove the background from the previous edges and the instrument. Finally, a quadratic polynomial was fitted to the post-edge region to normalize the spectra to the absorption of an isolated atom. Two regions in the $\mu(E)$ spectra are of interest, namely:

S4.1.1 XANES region

The X-ray absorption near edge structure (XANES) region is rich in information regarding the electronic state of the material. Representative normalized Fe K-edge spectra, collected at 300 K (RT) are compared to the parent β-FeSe in **Figure S11a,b**. The XANES show: (i) a pre-edge peak #A (~7112 eV), due to mixing of metal quadrupole 1*s* → 3*d* with ligand dipole 1*s* → 4*p* transitions, and (ii) above the edge, feature #B (~7119 eV), due to metal 1*s* → 4*p* transitions, admixed with the Se *d* states. The visible decrease in the intensity of #B in $Li_x(C_5H_5N)_yFe_2Se_2$, advising on the evolution of the Fe-site coordination chemistry[14] due to intercalation-induced charge-doping. On the other hand, the peak #A that depends on the oxidation state and local geometry,[15] shows a small diminution of the spectral weight upon intercalation (**Figure S11a**, inset). It implies a modest decrease in the Fe 3*d* and Se 4*p* orbitals mixing[16] due to $FeSe_4$ geometry modification (**Table 1**). In addition, on cooling through T_c, a somewhat steeper change of the peak #A intensity (**Figure S11c**), marks a reduced 3*d*–4*p* hybridization likely relevant to Hund's coupling mediated redistribution of 3*d* states in this multiorbital system.[17]

S4.1.2 EXAFS region:

While the XANES is limited to a few eVs around the edge, insights on the local lattice structure modifications are provided by the extended X-ray absorption fine structure (EXAFS) region, starting few tens of eV beyond the edge. **Figure S12** presents the temperature evolution of the normalised EXAFS oscillations and their corresponding Fourier Transforms for $Li_x(C_5H_5N)_yFe_2Se_2$. Modelling of the EXAFS oscillations was performed within the Artemis software suite.[13] The k^2-weighted EXAFS were analysed on the basis of the single scattering approximation,[18]

$$k^n\chi(k) = S_0^2 \sum_i N_i F_i(k_i) k_i^{n-1} e^{-2\sigma_i^2 k_i^2} e^{-\frac{2R_i}{\lambda_i(k_i)}} \times \frac{\sin[2k_i R_i + \varphi_i(k_i)]}{r_i^2} \quad (7)$$

where S_0^2 is the EXAFS reduction factor due to many-body effects. N_i is the number of neighbouring atoms at a distance R_i, $F_i(k_i)$ is the backscattering amplitude, k_i is the wave number of the photoelectron, σ_i^2 is the mean square relative displacements (MSRDs) of the photoabsorber-backscatterer pairs due to thermal motion and λ_i is the photoelectron mean free path. The φ_i is the phase shift implemented in the backscattered wave.

Fitting of EXAFS spectra was pursued by employing the crystal structure model of the tetragonal layered β-FeSe. Representative model fits, at 20 and 295 K are shown in **Figure 5**. Here, the Fe K-edge data were modelled by employing the 1st coordination shell, with 4 Se atoms at $R\sim$ 2.39 Å and the 2nd coordination shell, with 4 Fe atoms at $R\sim$ 2.67 Å. The two-shell model fits were performed in the k-range of 2.6 – 17.4 $Å^{-1}$ and R-range of 1.6 – 2.6 Å allowing for ~9 maximum number of independent fitted parameters (2 Δk $\Delta R/\pi$). In view of the two-shell model analysis, S_0^2 ≅ 0.652(8) and E_0 ≅ 2.1(2) eV were kept constant (taken as the average values evaluated over a broad temperature range), while the Fe-Se and Fe-Fe distances and the corresponding Debye-Waller factors (σ_i^2) were refined.

S4.1.3 EXAFS search for deviations from tetragonality

To investigate whether instantaneous atomic distortions that deviate from tetragonality may be witnessed in the intercalated phase, two scattering paths with respect to the Fe absorber were introduced for modeling of NN Fe-Fe distances, namely, $R_{1\_Fe\text{-}Fe}$= 2.672 Å, $R_{2\_Fe\text{-}Fe}$= 2.662 Å; such a geometry aligns with the orthorhombic *Cmma* symmetry, imposed by nematic fluctuations, as observed in β-FeSe[8]. By performing EXAFS fits at 20 K (and RT) we see no difference either in the quality of fit factors or at the metrics (MSRDs & distances). Essentially, when we try to refine a second Fe scattering path that is slightly shorter, we find that the distances [$R_{1\_Fe\text{-}Fe}$= $R_{2\_Fe\text{-}Fe}$= 2.670(8) Å] and the respective MSRDs [$\sigma^2_{\_Fe\text{-}Fe}$= 0.006(1) $Å^2$] converge to the value of the tetragonal model (**Figure S13**). This suggests that within the resolution of the present EXAFS data no discernible orthorhombicity can be found in the intercalated phase, consistent with the PDF findings.

Although there is no strict one-to-one correspondence between the magnitudes of the NN Fe-Se and Fe-Fe distances obtained from EXAFS and those derived from PDF analysis at 300 K, the comparison of the parent β-FeSe and the annealed sample (both measured during the same beamtime allocation) shows that EXAFS corroborates the PDF results at room temperature (**Table S3**). Specifically, the local geometrical parameters extracted from EXAFS follow the same trends observed in PDF when moving from β-FeSe to annealed phase: (i) elongation of the NN Fe-Se and Fe-Fe distances, (ii) compression of anion height, h_z, and (iii) widening of the bond angle, α.

S4.1.4 EXAFS and MSRDs

The MSRDs for a pair of atoms reflect the lattice configurational disorder expressed through the sum of temperature-independent (σ_0^2; static) and temperature-

dependent (σ_d^2; dynamic) terms, $\sigma^2 = \sigma_0^2 + \sigma_d^2(T)$.[19] The phenomenological correlated Einstein model is fitted against the MSRDs to extract information on the local bond dynamics. In this, model the T- dependent term is given,[19]

$$\sigma_d^2(T) = \frac{\hbar^2}{2\mu k_B \theta_{\mathrm{E}}} \coth\left(\frac{\theta_{\mathrm{E}}}{2T}\right) \tag{8}$$

where μ is the reduced mass of the respective atom pair and θ_{E} is the Einstein temperature, related to the Einstein frequency $\left(\omega_{\mathrm{E}} = \frac{k_B \theta_{\mathrm{E}}}{\hbar}\right)$, with the lattice force constant given as, $k = \mu\omega_E^2$. For the $Li_x(C_5H_5N)_yFe_2Se_2$ the analysis was carried down to 50 K. **Table S4** compares the lattice force constants after intercalation, with those of β-FeSe.[14] While the Fe-Fe network is not modified, the local Fe-Se bond becomes somewhat harder with respect to the parent. A similar response for the Fe-Se bond is also met in the expanded-lattice $(Li_{1-x}Fe_x)OHFeSe$ intercalation compound.[20] In $Li_x(C_5H_5N)_yFe_2Se_2$, the harder Fe-Se bond comes along with lower static disorder compared to that in the local Fe-Fe network (**Table S4**).

Figure S10. Basic sample characterization

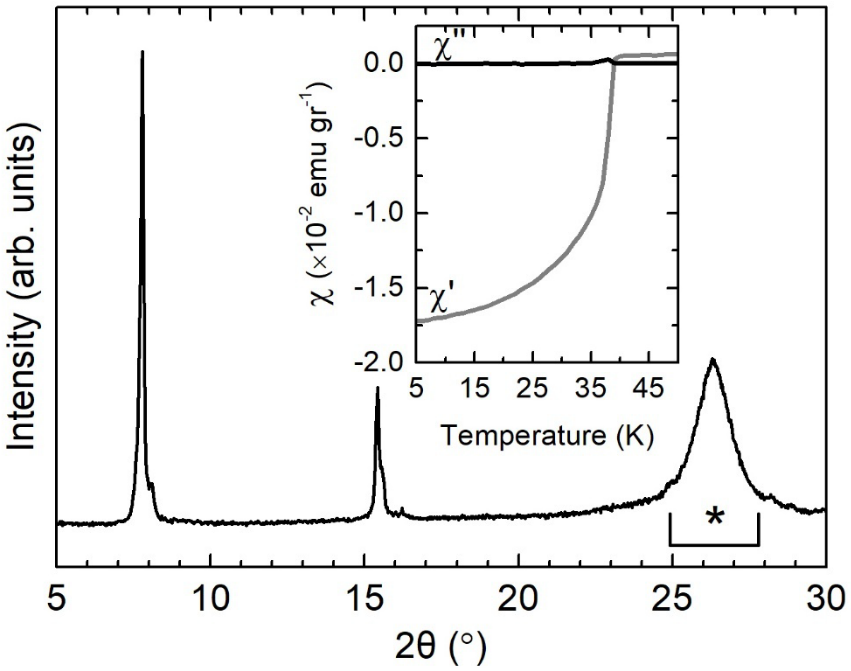

Figure S10. Powder X-ray diffraction (CuKα) pattern of the $Li_x(C_5H_5N)_yFe_2Se_2$ specimen used for the XAS and XES studies. The region corresponding to 2θ = 22 - 28° is a contribution (*) from the home-made, airtight sample holder. Inset: Normalized AC SQUID susceptibility (H_{ac} = 1 Oe and f = 499 Hz) of the real (χ'; gray line) and imaginary (χ''; black line) parts for the same sample.

Figure S11. Normalized XANES spectra

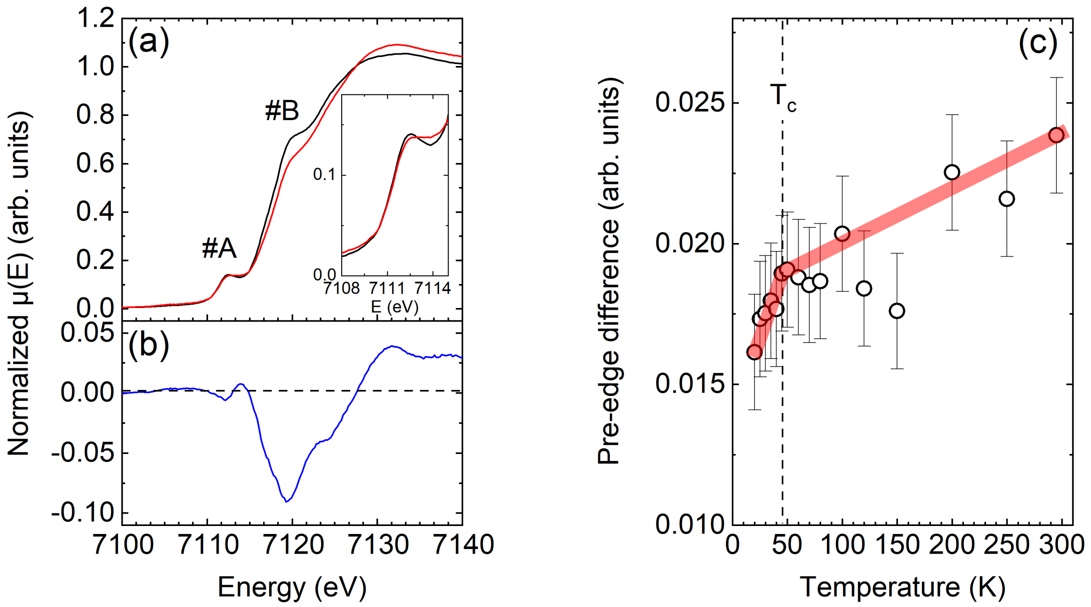

Figure S11. (a) Normalized XANES spectra (T= 20 K) at the Fe K edge of $Li_x(C_5H_5N)_yFe_2Se_2$ (red) compared to parent FeSe (black), and their (b) difference (blue). In part (a) the labels #A and #B identify near-edge spectra features. (c) Temperature evolution of the pre-edge peak #A intensity difference evaluated by subtracting the 295 K FeSe spectrum from the respective annealed spectrum, followed by integrating the absolute area in the energy range 7108 – 7113.7 eV. The thick red line is guide to the eye.

Figure S12. EXAFS oscillations and Fourier Transforms

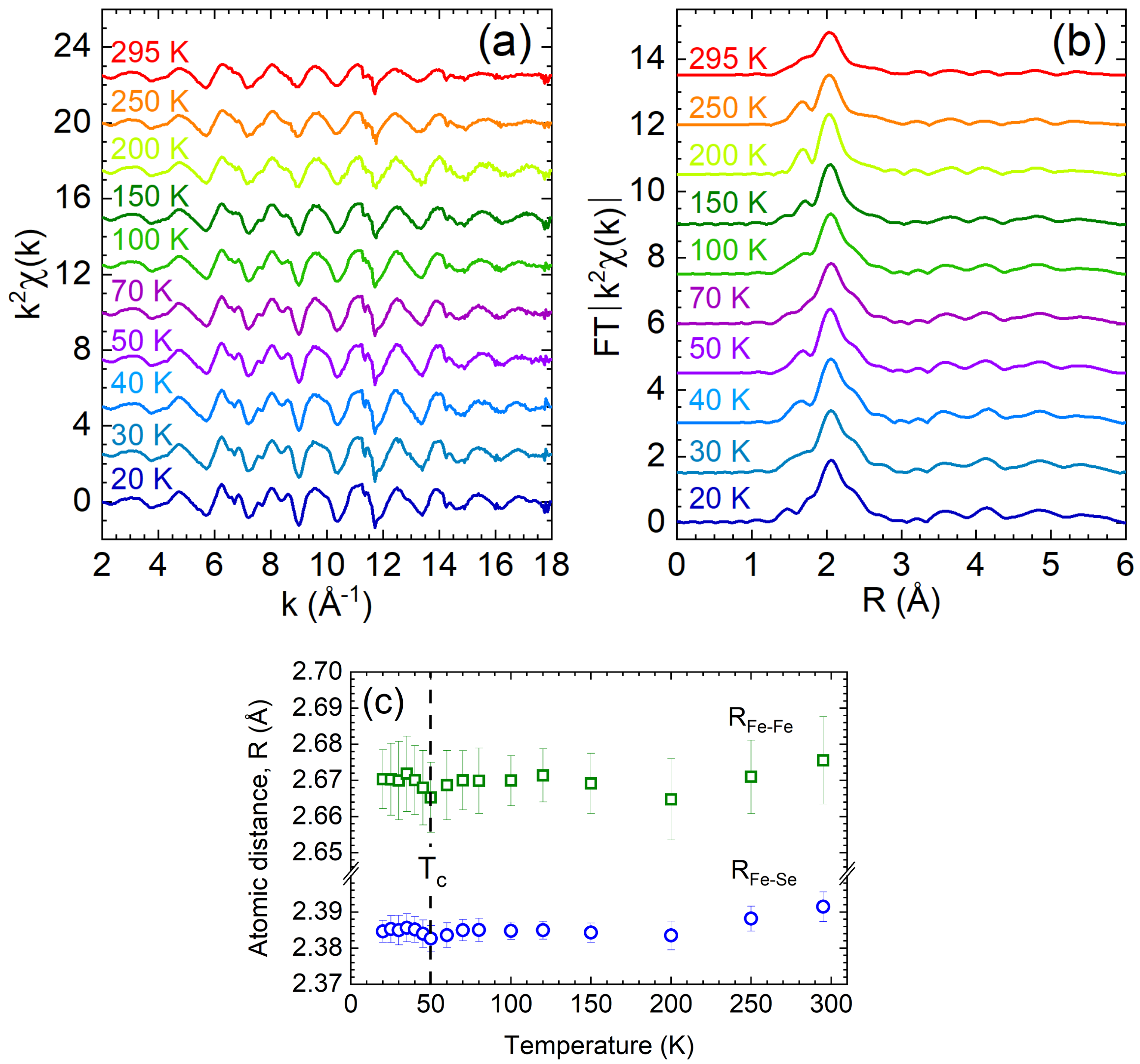

Figure S12. Temperature evolution of the (a) EXAFS oscillations and the corresponding (b) Fourier Transforms of the oscillations, in $Li_x(C_5H_5N)_yFe_2Se_2$ measured at the Fe K-edge. The spectra are shifted vertically for clarity. (c) Temperature evolution of the nearest neighbor Fe-Se (circles) and Fe-Fe (squares) atomic distances of the $Li_x(C_5H_5N)_yFe_2Se_2$ phase extracted from the two-shell model fit of the Fe K-edge EXAFS (see text §S4.1).

Figure S13. EXAFS search of deviations from tetragonality

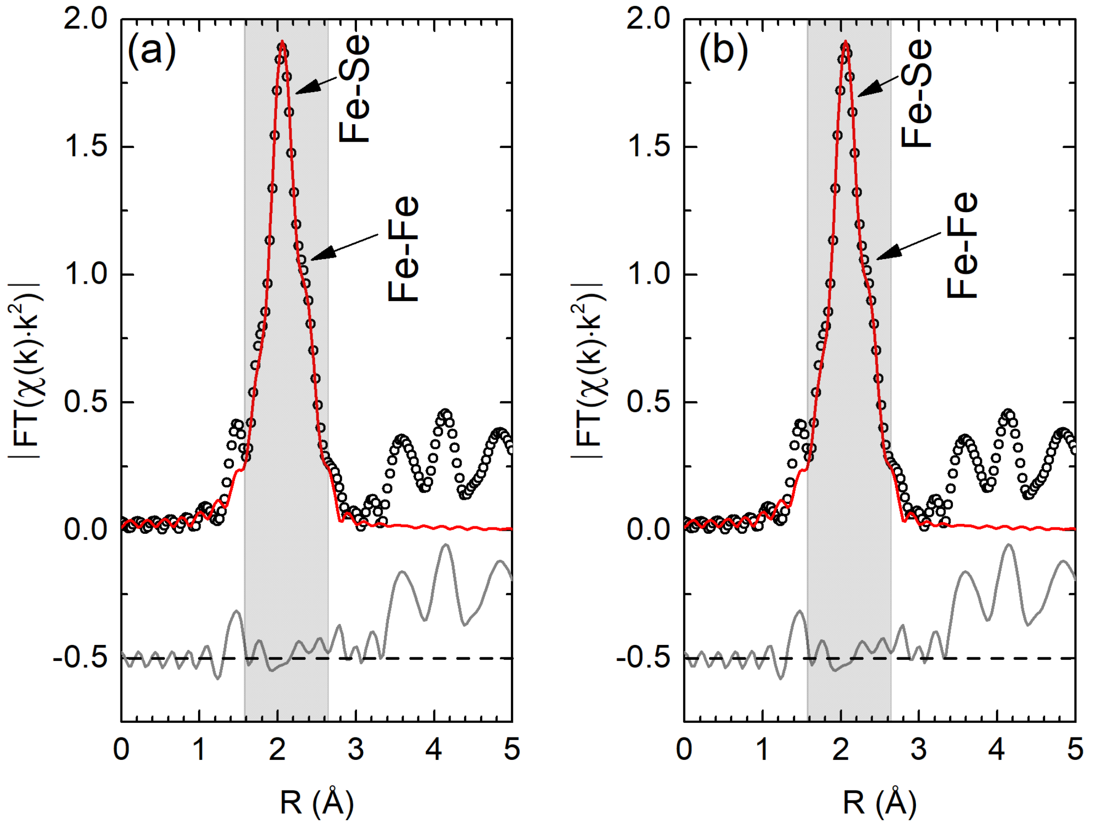

Figure S13. Representative two-shell model fits (red line) of the FT (circles) of the EXAFS oscillations at 20 K (chosen for its sharper radial distance features) for $Li_x(C_5H_5N)_yFe_2Se_2$. Their difference is also shown (gray line), while the highlighted region with the gray rectangle marks the R-region of the fit. The (a) tetragonal model fit employs a single scattering path while the (b) orthorhombic model fit employs two single scattering paths for the NN Fe-Fe neighbors.

Table S3. The nearest neighbor Fe-Se ($R_{Fe\text{-}Se}$), Fe-Fe ($R_{Fe\text{-}Fe}$) distances, the anion height (h_z) and bond angle (α) for the parent β-FeSe and $Li_x(C_5H_5N)_yFe_2Se_2$ tetragonal phases, derived from PDF and EXAFS analysis at 300 K.

	PDF		EXAFS	
	β-FeSe	$Li_x(C_5H_5N)_yFe_2Se_2$	β-FeSe	$Li_x(C_5H_5N)_yFe_2Se_2$
$R_{Fe\text{-}Se}$ (Å)	2.3876(1)	2.4162(4)	2.386(6)	2.391(4)
$R_{Fe\text{-}Fe}$ (Å)	2.6577(1)	2.7163(3)	2.66(1)	2.68(1)
h_z (Å)	1.471(3)	1.463(1)	1.47(1)	1.46(1)
α (°)	104.32(1)	105.18(3)	104.18(1)	104.58(1)

Table S4. The Einstein temperature (θ_{E}), lattice force constant (k) and static disorder MSRDs (σ_0^2), all extracted from the fit of the correlated Einstein model to the MSRDs of the $Li_x(C_5H_5N)_yFe_2Se_2$ compound, and compared with the respective parameters of the parent β-FeSe[14].

	β-FeSe		$Li_x(C_5H_5N)_yFe_2Se_2$	
	Fe-Se	Fe-Fe	Fe-Se	Fe-Fe
θ_{E} (K)	318 ± 5	263 ± 5	341 ± 8	259 ± 8 K
k (eV Å^{-2})	7.1(1)	3.4(1)	8.2(1)	3.3(1)
$\sigma_0^2(\text{Å}^2)$	0.00021(5)	0.00000(9)	0.00062(9)	0.0039(2)

S4.2 X-Ray Emission Spectroscopy: data analysis

In Fe-superconductors, interactions across different energy scale manifest in their magnetic response. The imaginary part of the local spin susceptibility, $\chi''(\boldsymbol{Q},\omega)$ provides a measure of how fast a system responds. This response may reflect (i) an instantaneous magnetic moment, at the short time limit, $\chi''(t\rightarrow 0)$ and/or (ii) a screened, static magnetic moment over longer timescales, $\chi''(t\rightarrow\infty)$.[21] While conventional magnetic measurements, such as NMR and Mössbauer spectroscopy are primarily sensitive to the latter, fundamental (sub-picosecond) processes that occur in inelastic neutron scattering (INS) and hard X-ray spectroscopies are more suitable for capturing behaviors at faster timescales. Accordingly, X-ray emission spectroscopy (XES), a fast probe with local sensitivity on the timescale of electron dynamics (*fs*), has been employed as it plays a crucial role in exploring such phenomena. In particular, the Fe K*β* emission spectrum (**Figure S14**) offers a direct probe of the local Fe magnetic moment (μ) manifesting at short timescales.[22,23]

To this end, the integrated absolute difference (IAD) method is employed to quantitatively extract the Fe magnetic moment (μ) information from Fe K*β* emission spectra by comparison with a non-magnetic reference.[22] The procedure for IAD extraction involves: (i) normalization of the spectra by their integrated area, (ii) subtraction of the reference spectrum from each dataset and (iii) integration of the absolute area of the resulting difference spectra (**Figure S15** and **Figure 6**). The obtained IAD value is proportional to the magnitude of the local Fe μ. In the low-resolution dataset (~4.0 eV), the temperature evolution ($20 \leq T \leq 295$ K) of the IAD for the $Li_x(C_5H_5N)_yFe_2Se_2$ system (**Figure 7a**) is determined using the 100 K spectrum of the parent β-FeSe as the reference. Assuming that fundamental processes render INS a fast technique (cf., ~10^{-14} s, but not as fast as XES), considering the lack of a spin-resonance in recent INS experiments[24] with the evolution of IAD in $Li_x(C_5H_5N)_yFe_2Se_2$ that begins to increase near T_S, may not waive out rapidly fluctuating spin moments in this system at $T< T_S$.

Additional evaluations between the 20 K and 295 K spectra of $Li_x(C_5H_5N)_yFe_2Se_2$, at both high and low spectral resolutions, were conducted by aligning the corresponding room temperature spectrum to the center of gravity (COG) of the 20 K spectrum (**Figure S14**). This alignment to the COG calibrates the spectra to an absolute energy scale,[23] a method previously applied in studies of Fe K*β* emission lines of other Fe-based superconductors.[25]

Figure S14. Normalized XES spectra: high- *vs*. low- spectral resolutions

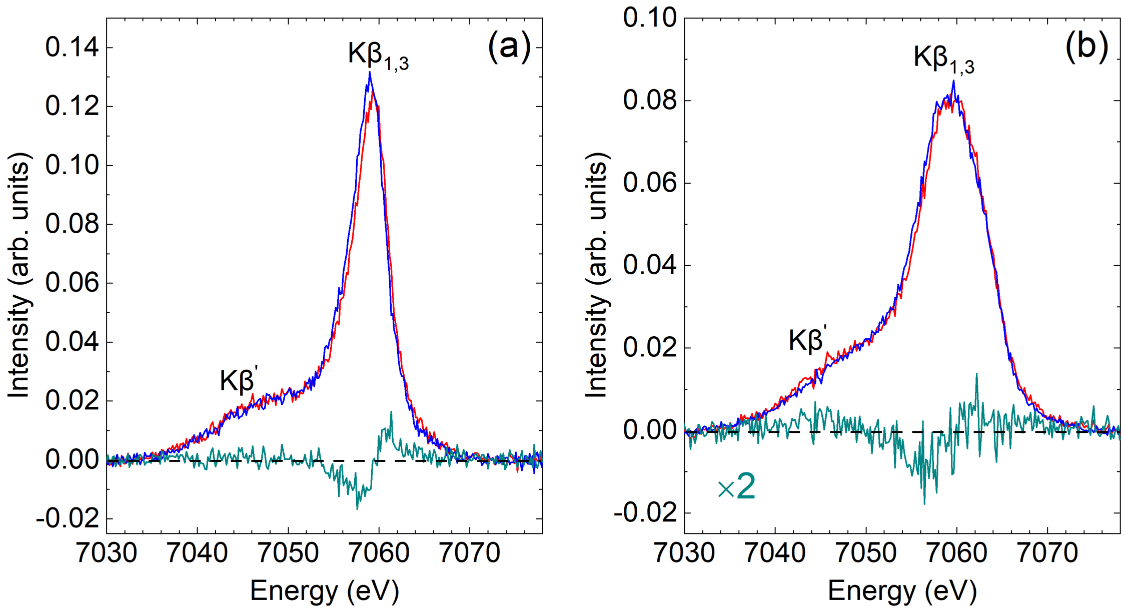

Figure S14. XES spectra of $Li_x(C_5H_5N)_yFe_2Se_2$ measured at 295 K (red) and 20 K (blue) along with their respective difference (295 K – 20 K; cyan). The spectra were recorded with energy resolution of (a) 1.5 eV and (b) 4.0 eV. The 295 K spectra have been aligned to the COG of the 20 K spectra. In part (b) the difference line is multiplied by two.

Figure S15. Normalized XES spectra: temperature evolution

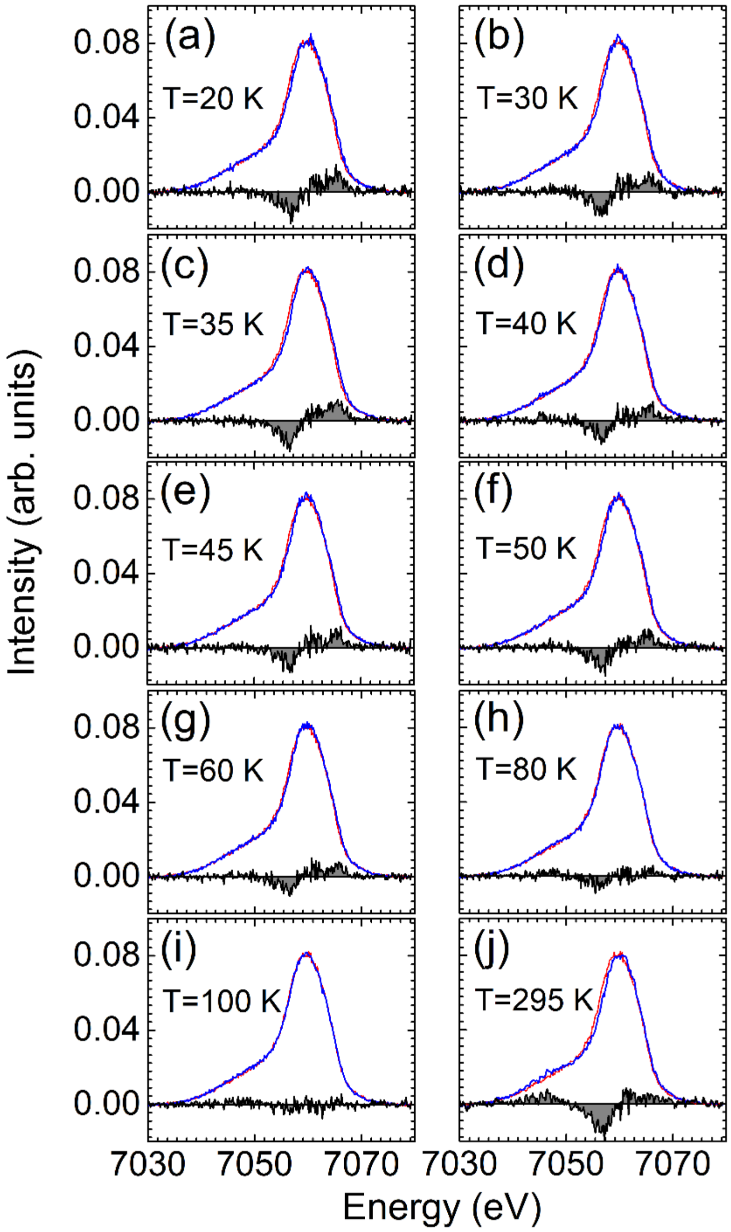

Figure S15. (a) XES spectra of $Li_x(C_5H_5N)_yFe_2Se_2$ recorded with energy resolution of 4.0 eV, as a function of temperature. (b)-(k) The XES of $Li_x(C_5H_5N)_yFe_2Se_2$ (blue) and the reference β-FeSe (red) along with their respective difference (black). The spectral difference curves are multiplied by two.

S5. References

(1) Toby, B. H.; Von Dreele, R. B. *GSAS-II* : The Genesis of a Modern Open-Source All Purpose Crystallography Software Package. *J. Appl. Crystallogr.* **2013**, *46* (2), 544–549. https://doi.org/10.1107/S0021889813003531.

(2) Coelho, A. A. TOPAS and TOPAS-Academic: An Optimization Program Integrating Computer Algebra and Crystallographic Objects Written in C++. *J. Appl. Crystallogr.* **2018**, *51* (1), 210–218. https://doi.org/10.1107/S1600576718000183.

(3) Holy, N. L. Reactions of the Radical Anions and Dianions of Aromatic Hydrocarbons. *Chem. Rev.* **1974**, *74* (2), 243–277. https://doi.org/10.1021/cr60288a005.

(4) Melero, C.; Guijarro, A.; Yus, M. Structural Characterization and Bonding Properties of Lithium Naphthalene Radical Anion, $[Li^+(TMEDA)_2][C_{10}H_8^{\cdot-}]$, and Lithium Naphthalene Dianion $[(Li^+TMEDA)_2C_{10}H_8^{-2}]$. *Dalton Trans.* **2009**, 8, 1286–1289. https://doi.org/10.1039/B821119C.

(5) Kang, H. S. Density Functional Study of Lithium−Aromatic Sandwich Compounds and Their Crystals. *J. Phys. Chem. A* **2005**, *109* (3), 478–483. https://doi.org/10.1021/jp0464876.

(6) Morton-Blake, D. A.; Corish, J.; Bénière, F. A Molecular Orbital Study of the Bonding in Complexes of Lithium with Polynuclear Aromatic Hydrocarbons. *Theor. Chim. Acta* **1985**, *68* (5), 389–405. https://doi.org/10.1007/BF00529059.

(7) Burrard-Lucas, M.; Free, D. G.; Sedlmaier, S. J.; Wright, J. D.; Cassidy, S. J.; Hara, Y.; Corkett, A. J.; Lancaster, T.; Baker, P. J.; Blundell, S. J.; Clarke, S. J. Enhancement of the Superconducting Transition Temperature of FeSe by Intercalation of a Molecular Spacer Layer. *Nat. Mater.* **2013**, *12* (1), 15–19. https://doi.org/10.1038/nmat3464.

(8) Koch, R. J.; Konstantinova, T.; Abeykoon, M.; Wang, A.; Petrovic, C.; Zhu, Y.; Bozin, E. S.; Billinge, S. J. L. Room Temperature Local Nematicity in FeSe Superconductor. *Phys. Rev. B* **2019**, *100* (2), 020501. https://doi.org/10.1103/PhysRevB.100.020501.

(9) McQueen, T. M.; Williams, A. J.; Stephens, P. W.; Tao, J.; Zhu, Y.; Ksenofontov, V.; Casper, F.; Felser, C.; Cava, R. J. Tetragonal-to-Orthorhombic Structural Phase Transition at 90 K in the Superconductor $Fe_{1.01}Se$. *Phys. Rev. Lett.* **2009**, *103* (5), 057002. https://doi.org/10.1103/PhysRevLett.103.057002.

(10) Stephens, P. W. Phenomenological Model of Anisotropic Peak Broadening in Powder Diffraction. *J. Appl. Crystallogr.* **1999**, *32* (2), 281–289. https://doi.org/10.1107/S0021889898006001.

(11) Mizuguchi, Y.; Hara, Y.; Deguchi, K.; Tsuda, S.; Yamaguchi, T.; Takeda, K.; Kotegawa, H.; Tou, H.; Takano, Y. Anion Height Dependence of T_c for the Fe-Based Superconductor. *Supercond. Sci. Technol.* **2010**, *23* (5), 054013. https://doi.org/10.1088/0953-2048/23/5/054013.

(12) M. Kaitatzi, A. Deltsidis, A. Lappas, et al. *Unpublished Results*. **2025**.

(13) Ravel, B.; Newville, M. *ATHENA* , *ARTEMIS* , *HEPHAESTUS* : Data Analysis for X-Ray Absorption Spectroscopy Using *IFEFFIT*. *J. Synchrotron Radiat.* **2005**, *12* (4), 537–541. https://doi.org/10.1107/S0909049505012719.

(14) Deltsidis, A.; Simonelli, L.; Vailakis, G.; Berdiell, I. C.; Kopidakis, G.; Krztoń-Maziopa, A.; Bozin, E. S.; Lappas, A. $Li_x(C_5H_5N)_yFe_{2-z}Se_2$: A Defect-Resilient Expanded-Lattice High-Temperature Superconductor. *Inorg. Chem.* **2022**, *61* (32), 12797–12808. https://doi.org/10.1021/acs.inorgchem.2c01906.

(15) Wong, J.; Lytle, F. W.; Messmer, R. P.; Maylotte, D. H. K -Edge Absorption Spectra of Selected Vanadium Compounds. *Phys. Rev. B* **1984**, *30* (10), 5596–5610. https://doi.org/10.1103/PhysRevB.30.5596.

(16) Westre, T. E.; Kennepohl, P.; DeWitt, J. G.; Hedman, B.; Hodgson, K. O.; Solomon, E. I. A Multiplet Analysis of Fe K-Edge 1s → 3d Pre-Edge Features of Iron Complexes. *J. Am. Chem. Soc.* **1997**, *119* (27), 6297–6314. https://doi.org/10.1021/ja964352a.

(17) Yi, M.; Liu, Z.-K.; Zhang, Y.; Yu, R.; Zhu, J.-X.; Lee, J. J.; Moore, R. G.; Schmitt, F. T.; Li, W.; Riggs, S. C.; Chu, J.-H.; Lv, B.; Hu, J.; Hashimoto, M.; Mo, S.-K.; Hussain, Z.; Mao, Z. Q.; Chu, C. W.; Fisher, I. R.; Si, Q.; Shen, Z.-X.; Lu, D. H. Observation of Universal Strong Orbital-Dependent Correlation Effects in Iron Chalcogenides. *Nat. Commun.* **2015**, *6* (1), 7777. https://doi.org/10.1038/ncomms8777.

(18) Teo, B. K. *EXAFS: Basic Principles and Data Analysis*; Jørgensen, C. K., Lappert, M. F., Lippard, S. J., Margrave, J. L., Niedenzu, K., Nöth, H., Parry, R. W., Yamatera, H., Series Eds.; Inorganic Chemistry Concepts; Springer Berlin Heidelberg: Berlin, Heidelberg, **1986**; Vol. 9. https://doi.org/10.1007/978-3-642-50031-2.

(19) Rehr, J. J.; Albers, R. C. Theoretical Approaches to X-Ray Absorption Fine Structure. *Rev. Mod. Phys.* **2000**, *72* (3), 621–654. https://doi.org/10.1103/RevModPhys.72.621.

(20) Tomassucci, G.; Tortora, L.; Pugliese, G. M.; Stramaglia, F.; Simonelli, L.; Marini, C.; Terashima, K.; Wakita, T.; Ayukawa, S.; Yokoya, T.; Kudo, K.; Nohara, M.; Mizokawa, T.; Saini, N. L. Temperature Dependent Local Inhomogeneity and Magnetic Moments of $(Li_{1-x}Fe_x)OHFeSe$ Superconductors. *Phys. Chem. Chem. Phys.* **2023**, *25* (9), 6684–6692. https://doi.org/10.1039/D3CP00004D.

(21) Mannella, N. The Magnetic Moment Enigma in Fe-Based High Temperature Superconductors. *J. Phys. Condens. Matter* **2014**, *26* (47), 473202. https://doi.org/10.1088/0953-8984/26/47/473202.

(22) Vankó, G.; Neisius, T.; Molnár, G.; Renz, F.; Kárpáti, S.; Shukla, A.; de Groot, F. M. F. Probing the 3d Spin Momentum with X-Ray Emission Spectroscopy: The Case of Molecular-Spin Transitions. *J. Phys. Chem. B* **2006**, *110* (24), 11647–11653. https://doi.org/10.1021/jp0615961.

(23) Glatzel, P.; Bergmann, U. High Resolution 1s Core Hole X-Ray Spectroscopy in 3d Transition Metal Complexes—Electronic and Structural Information. *Coord. Chem. Rev.* **2005**, *249* (1), 65–95. https://doi.org/10.1016/j.ccr.2004.04.011.

(24) Deltsidis, A.; Kaitatzi, M.; Simonelli, L.; Stock, C.; Voneshen, D.; Lappas, A. Local Structure and Phonon States Mediated by Intercalation-Driven Doping in Superconducting $Li_{1.0}(C_5H_5N)_yFe_{2-z}Se_2$. *Phys. Rev. Mater.* **2025**, *9* (2), 024801. https://doi.org/10.1103/PhysRevMaterials.9.024801.

(25) Pelliciari, J.; Ishii, K.; Huang, Y.; Dantz, M.; Lu, X.; Olalde-Velasco, P.; Strocov, V. N.; Kasahara, S.; Xing, L.; Wang, X.; Jin, C.; Matsuda, Y.; Shibauchi, T.; Das, T.; Schmitt, T. Reciprocity between Local Moments and Collective Magnetic Excitations in the Phase Diagram of $BaFe_2(As_{1-x}P_x)_2$. *Commun. Phys.* **2019**, *2* (1), 139. https://doi.org/10.1038/s42005-019-0236-3.
